# Transcriptomic analyses and leukocyte telomere length measurement in subjects exposed to severe recent stressful life events

**DOI:** 10.1038/tp.2017.5

**Published:** 2017-02-21

**Authors:** N Lopizzo, S Tosato, V Begni, S Tomassi, N Cattane, M Barcella, G Turco, M Ruggeri, M A Riva, C M Pariante, A Cattaneo

**Affiliations:** 1 Biological Psychiatry Unit, IRCCS Fatebenefratelli S. Giovanni di Dio, Brescia, Italy; 2 Department of Neurosciences, Biomedicine and Movement Sciences, Section of Psychiatry, University of Verona, Verona, Italy; 3 Department of Pharmacological and Biomolecular Sciences, University of Milan, Milan, Italy; 4 Stress, Psychiatry and Immunology Laboratory, Department of Psychological Medicine, Institute of Psychiatry, King's College, London, London, UK

## Abstract

Stressful life events occurring in adulthood have been found able to affect mood and behavior, thus increasing the vulnerability for several stress-related psychiatric disorders. However, although there is plenty of clinical data supporting an association between stressful life events in adulthood and an enhanced vulnerability for psychopathology, the underlying molecular mechanisms are still poorly investigated. Thus, in this study we performed peripheral/whole-genome transcriptomic analyses in blood samples obtained from 53 adult subjects characterized for recent stressful life events occurred within the previous 6 months. Transcriptomic data were analyzed using Partek Genomics Suite; pathway and network analyses were performed using Ingenuity Pathway Analysis and GeneMANIA Software. We found 207 genes significantly differentially expressed in adult subjects who reported recent stressful life experiences (*n*=21) compared with those without such experiences (*n*=32). Moreover, the same subjects exposed to such stressful experiences showed a reduction in leukocyte telomere length. A correlation analyses between telomere length and transcriptomic data indicated an association between the exposures to recent stressful life events and the modulation of several pathways, mainly involved in immune-inflammatory-related processes and oxidative stress, such as natural killer cell signaling, interleukin-1 (IL-1) signaling, MIF regulation of innate immunity and IL-6 signaling. Our data suggest an association between exposures to recent stressful life events in adulthood and alterations in the immune, inflammatory and oxidative stress pathways, which could be also involved in the negative effect of stressful life events on leukocyte telomere length. The modulation of these mechanisms may underlie the clinical association between the exposure to recent Stressful life events in adulthood and an enhanced vulnerability to develop psychiatric diseases in adulthood.

## Introduction

Stressful life events (SLEs) occurring in adulthood, such as illnesses, social difficulties, unemployment, and loss of an intimate relationship because of death or separation, not only impair the quality of life of an individual,^1^ but also increase the risk for developing both physical and mental disorders, such as metabolic syndromes,^2^ cardiovascular diseases,^3^ post-traumatic stress disorder,^4, 5^ major depression^6, 7^ and bipolar disorder.^8, 9^ Most of the evidence supporting these relationships have focused the attention on short-term consequences of stress, typically within a period of no >1 year.^10, 11^ Importantly, it has been reported that severe recent SLEs are impactful on the onset of psychiatric disorders,^12^ in particular, the initial episodes are more likely to be precipitated by a severe SLE experience.^12, 13^ The biological responses to SLEs can occur mainly via the involvement of two different biological systems, both responsive to stress, the sympathetic–adrenal–medullary axis and the hypothalamic–pituitary–adrenal axis, thereby inducing the release of pituitary and adrenal hormones. Indeed, adrenaline and noradrenaline, adrenocorticotropic hormone, cortisol, growth hormone and prolactin are all influenced by SLEs, and each of them can induce alterations in immune functions.^14, 15, 16^ This immune modulation might occur directly, through the binding of the hormone to its receptor or indirectly, by inducing alterations in the production of cytokines.^17^ The capability of SLEs to modulate immune system has been supported by a recent meta-analysis, which shows a significant association between exposure to SLEs in the pre-diagnostic period and the development of several autoimmune diseases, like rheumatoid or psoriatic arthritis, type 1 diabetes, multiple sclerosis and autoimmune thyroid disease, suggesting that these kinds of stressors can have a key role in the etiopathogenesis of a wide range of diseases, all characterized by immune system alterations.^18^ Recently, it has been suggested that SLEs can also be involved in the mechanisms underlying telomere shortening.^19, 20, 21^ Telomeres are DNA–protein complexes at the end of chromosomes, composed of tandem TTAGGG repeats ranging from a few to 15 kb in length. Telomeres are essential for providing protection from enzymatic degradation and for maintaining chromosomal stability, therefore, an adequate telomere structure is pivotal in avoiding cellular dysfunctions. Telomeres shorten in each cell division, and the maintenance of their functions depends both on a minimal length of TTAGGG repeats and on the presence of telomere-binding proteins.^22^ Telomere length decreases physiologically during aging, but this shortening can be accelerated by a combination of genetic, epigenetic and environmental factors.^23, 24, 25^ Importantly, telomere shortening can also be influenced by alterations in the immune and stress response systems^26, 27^ and, in support to this, leukocyte telomere length have been found altered in patients with chronic inflammation,^28^ with mood disorders^29, 30^ and also in subjects exposed to chronic social stress or in post-traumatic stress disorder patients exposed to childhood trauma.^31, 32^ In a recent meta-analysis, Darrow *et al.*^33^ examined the relationship between cellular aging as indicated by leukocyte telomere length and a wide range of psychiatric disorders, including 14 827 participants from several studies, supporting the hypothesis that shortened leukocyte telomere length is seen across many psychiatric disorders, with a larger effect size in post-traumatic stress disorder, anxiety disorders and depressive disorders as compared with psychotic and bipolar disorder that had smaller, but significant, effect size. These data were partially in contrast with a recent systematic review and meta-analysis by Colpo *et al.*,^34^ where they reported no significant difference of telomere length in bipolar disorder patients as compared with control subjects. These contrasting data suggest that the relationship between psychiatric disorders and telomere length is not yet completely clear and needs further investigation.

Although plenty of literature data have supported the relationship between exposure to recent SLEs in adulthood and alterations in mood and behavior,^1, 9^ the biological mechanisms underlying this association are still not clear. Thus, in this study, in order to identify possible pathways and networks influenced by recent SLEs and potentially involved in the vulnerability for psychiatric disorders, we have used a hypothesis-free approach and assessed the entire transcriptome in the peripheral blood of adult subjects exposed to one or more SLEs in the previous 6 months. Moreover, we have investigated possible associations between the identified biological pathways influenced by SLEs and leukocyte telomere length.

## Materials and methods

### Participants and clinical assessment

Healthy subjects were recruited through notices posted at the Verona University Hospital, Verona (Italy). Individuals presenting a history of neurological or psychiatric diseases, prior traumatic brain injury, or mental retardation (IQ<70) were excluded from the study. The absence of psychiatric disorder was ascertained via two schedules: the Mini International Neuropsychiatric Interview (M.I.N.I. Plus^35^), to exclude any psychiatric disorder in Axis I; and the Structured Clinical Interview for DSM disorders (SCID-II^36^) to exclude any psychiatric disorder in Axis II. Moreover, depressive symptoms were evaluated by the administration of the Hamilton Rating Scale for Depression (HAMD)^37^ and symptoms of mania by the administration of the Bech-Rafaelsen Mania Rating Scale (BRMRS).^38^

In addition, being pregnant or in lactation represented an exclusion criterion. Subjects underwent also a detailed medical examination including tobacco and current drug therapies. Written informed consent was obtained by participants after receiving a complete description of the study, which has been approved by the ethic committee of the Verona University Hospital. All the subjects were assessed for exposures to childhood traumatic experiences, by the CECA-Q scale,^39^ and in adulthood by the administration of a modified version of the Life Events Scale.^40^ The latter one is a 56-item instrument and it covers a comprehensive range of recent life events, their timing and their date. It has two time frames for evaluation: (1) life events that occurred during the 6 months before the assessment; (2) those that occurred before the last 6 months (lifetime). As our specific aim was to identify possible short-term consequences of stress on pathways and networks, we focused only on recent SLEs (that is, ⩽ 6 months).

The severity of each SLE was assessed using the Holmes–Rahe Life Stress Inventory.^41^ On the basis of our previous work,^42^ only ‘severe stressful life events' (that is, death of a family member, sexual or physical abuse, being accused of having committed a crime, sentence of imprisonment, being exposed to war and natural catastrophes, family breakdown, being removed from home, sentimental breakdown and severe physical illness) were taken into account.

According to inclusion criteria, we recruited and assessed a total of 72 subjects. Out of these, 8 subjects reported both childhood trauma and SLEs experiences, 11 subjects reported childhood trauma only, 32 subjects reported neither childhood trauma nor recent adulthood traumatic experiences and 21 subjects reported SLEs but not childhood trauma. As we wanted to evaluate specifically the short-term effects of stress in adulthood, excluding any possible long-lasting effect due to childhood trauma exposures, we focused the attention on the group of 32 subjects who reported neither severe SLEs nor childhood trauma and on the 21 subjects that experienced at least one severe recent SLEs in adulthood, but not childhood trauma.

Blood samples were collected fasting in the morning by using PaxGene Blood RNA Tubes (PreAnalytix, Hombrechtikonas, Switzerland) and BD Vacutainer K2E EDTA Tubes (BD-Plymouth, Oxford, UK) for DNA isolation and in anticoagulant-free tubes for serum (BD-Plymouth).

Subjects exposed and subjects not exposed to SLEs were similar for age, gender, smoking, body mass index, depressive symptoms (in term of HAMD scores), ethnicity, education and marital status (Supplementary Table 1). Moreover, the two study groups were not significantly different for the presence of recent drug therapies (subjects exposed to SLEs, *n*=21: 2/21 were receiving drugs for thyroid disorders, 5/21 were receiving cortisonic drugs, 2/21 were receiving psychotropic drugs, 1/21 was receiving drugs for gastrointestinal disorders, 2/21 were receiving drugs for cardiovascular diseases; subjects not exposed to SLEs, *n*=32: 2/32 were receiving drugs for thyroid disorders, 2/32 were receiving cortisonic drugs, 3/32 were receiving psychotropic drugs, 2/32 were receiving drugs for gastrointestinal disorders, 1/32 was receiving drugs for cardiovascular diseases).

### RNA and DNA isolation

After blood collection, PaxGene Tubes were kept at room temperature for 2 h, then at −20 °C for 2 days and then at −80 °C until their processing for RNA isolation. Total RNA was isolated using PaxGene miRNA kit (Qiagen, Hilden, Germany) according to the manufacturer's instructions and RNA quantity and quality was assessed by evaluation of the A260/280 and A260/230 ratios using a Nanodrop spectrophotometer (NanoDrop Technologies, Wilmington, DE, USA). Genomic DNA was isolated from peripheral whole blood, by using the Gentra Puregene Blood Kit (Qiagen), according to the manufacturer's instructions and DNA quality was assessed by evaluation of the A260/280 and A260/230 ratios using a Nanodrop spectrophotometer (NanoDrop Technologies).

### Serum cortisol determination

After blood sample collection, anticoagulant-free tubes have been kept at room temperature for 2 h, followed by 1 hour at 4 °C before serum separation by centrifugation (1620 g for 15 min) and subsequently, serum samples were kept at −80 °C until the time of the assay. Cortisol levels were measured by enzyme-linked immunosorbent assay (ELISA) method using the Human Cortisol Quantikine ELISA Kit (R&D Systems, Minneapolis, MN, USA), according to the manufacturer's instructions; the minimal detection limit for serum cortisol, by using this kit, is 0.071 ng ml^−1^. The optical density was recorded at 450 nm wavelength with an automated ELISA-plate reader and subsequently the absorbance was converted to ng/ml for cortisol. All the samples were evaluated in duplicate together with the standard curve.

### Whole-genome expression analyses

Gene expression microarray assays were performed as reported in our previous works,^43, 44^ using Human Gene 1.1 ST Array Strips on GeneAtlas platform (Affymetrix, Wycombe, UK) and following the WT Expression Kit protocol described in the Affymetrix GeneChip Expression Analysis Technical Manual. Briefly, 250 ng RNA were used to synthesize second strand cDNA with the Ambion Express Kit (Ambion, Life Technologies, Monza, Italy) and subsequently, the purified cDNA was fragmented and hybridized onto Human Gene 1.1 ST Array strips. The reactions of hybridization, fluidics and imaging were performed on the Affymetrix Gene Atlas instrument according to the manufacturer's protocol.

### Telomere length measurement by quantitative real-time PCR

Leukocyte telomere length was measured using the quantitative real-time PCR method. A six-point standard curve, derived from serially diluted DNA pool and ranging from 50 to 1.25 ng μl^−1^, was included in each PCR plate, so that relative quantities of telomere repeat (T) and single-copy gene number (S) could be determined. All the samples were run in triplicate on a CFX 384 Real-Time PCR System (Bio-Rad, Milan, Italy). Data were calculated by the method described by Cawthon^45^ that measures the relative telomere length in genomic DNA by determining the T/S ratio. The data were then expressed in term of Relative Expression Ratio, with subjects not exposed to recent SLEs as control group.

### Statistical and bioinformatic analysis

Data (mean±s.d. or s.e.m.) were analyzed using the Statistical Package for Social Sciences, Version 22.0 (SPSS).

For comparison of demographical and clinical variables between groups, student's t-test or chi-square-test were applied.

For transcriptomic analyses, Affymetrix CEL files were imported into Partek Genomics Suite (version 6.6) for data visualization, quality control assessment and statistical testing.

Quality criteria for hybridization controls, labeling controls and 3′/5′ Metrics have been passed by all the samples. Background correction was conducted using Robust Multi-strip Average (RMA)^46^ to remove noise from auto fluorescence. After background correction, normalization was conducted using Quantiles Normalization^47^ to normalize the distribution of probe intensities among different microarray chips. Subsequently, a summarization step was conducted using a linear median polish algorithm^48^ to integrate probe intensities in order to compute the expression levels for each gene transcript. After having performed a quality control of the data, analysis of the variance (ANOVA) test was performed to assess the effect of recent SLEs, comparing subjects exposed to SLEs vs subjects not exposed. List of significant genes was then obtained by applying a fold change (FC) cutoff of 10% and by applying a multiple testing correction procedure for all our data (*q*-value<0.05).

Lists of significant genes were then uploaded in Ingenuity Pathway Analyses Software to identify molecular pathways associated to recent SLEs exposure and pathways with a cutoff of *P*-value<0.05 were considered significant.

For network analyses we used the tool Gene Multiple Association Network Integration Algorithm (GeneMANIA) (http://www.genemania.org/), which is a web-based tool for the prediction of gene function. Based on single gene or gene set query from 7 organisms, it shows results for interactive functional associative network according to their co-expression data from Gene Expression Omnibus (GEO), physical and genetic interaction data derived from BioGRID, predicted protein interaction data based on orthology from I2D, co-localization, shared protein domain, and GO function.^49^

Cortisol levels and telomere length data were shown as mean±s.e.m. and, as their distribution was normal after a Kolmogorov–Smirnov test (*P*>0.05), we run Univariate General Linear Model, using age and gender whenever appropriate.

Correlation analyses between transcriptomics data and leukocyte telomere length, HAMD score, peripheral blood cortisol levels were run by using a Pearson correlation analyses; correlation analyses between telomere length or cortisol levels with smoking, gender, age were run by applying a Spearman correlation analyses.

## Results

### Transcriptomic analyses in the blood of subjects characterized for recent SLEs exposure

Our first aim was to identify differences in gene expression levels in association with recent SLEs exposure, thus we conducted a transcriptome analysis in the blood of subjects exposed (*n*=21) and not exposed (*n*=32) to recent SLEs and we found, comparing the two groups, significant differences in the expression of 207 genes. We listed the 24 top modulated genes (12 upregulated and 12 downregulated) in Table 1, and all the significant genes in the Supplementary Table 2. We then performed a pathway analyses on the 207 genes and we found 38 significantly modulated pathways (*P*<0.05) in association with recent SLEs exposures. Among the most significant biological processes there are several pathways involved in the modulation of the immune system and inflammation (including natural killer cell signaling, T-cell receptor signaling, crosstalk between dendritic cells and natural killer cells) and metabolism (including superpathway of methionine degradation, glycine biosynthesis I). The entire list of significant pathways is shown in Table 2.

We performed correlation analyses between the transcriptome profile and the HAMD scores and we identified a list of 201 significantly correlated genes (*P*<0.05; Supplementary Table 3). Among the top significant genes, we found several genes that have been found associated with mood disorders such as period circadian clock 1 (PER1),^50, 51^ interferon alpha 2 (IFNA2)^52^ and period circadian clock 2 (PER2).^53^

### Cortisol levels analysis

Here we wanted to evaluate possible differences in the stress response system in association with exposure to recent and severe SLEs, thus we assessed cortisol levels in the serum of subjects exposed and in those not exposed to SLEs. Cortisol levels did not correlate with any of the demographic features as well as with HAMD score or telomere length (all the *P*-values >0.05).

We didn't find any significant differences in cortisol levels in the two groups (mean±s.e.m.: 15.88±1.73 vs 18.97±1.58 ng ml^−1^ in subjects exposed vs subjects not exposed to SLEs, respectively; *P*=0.22). Correlation analyses between cortisol levels and transcriptomic profile identified 321 significantly correlated genes (Supplementary Table 4) and among the top significant ones we found several genes that have been found associated with inflammation and stress response such as CD69 molecule (CD69),^54^ period circadian clock 1 (PER1)^55^ and chemokine receptor type 4 (CXCR4).^56^

### Network analysis

**Figure 1:**
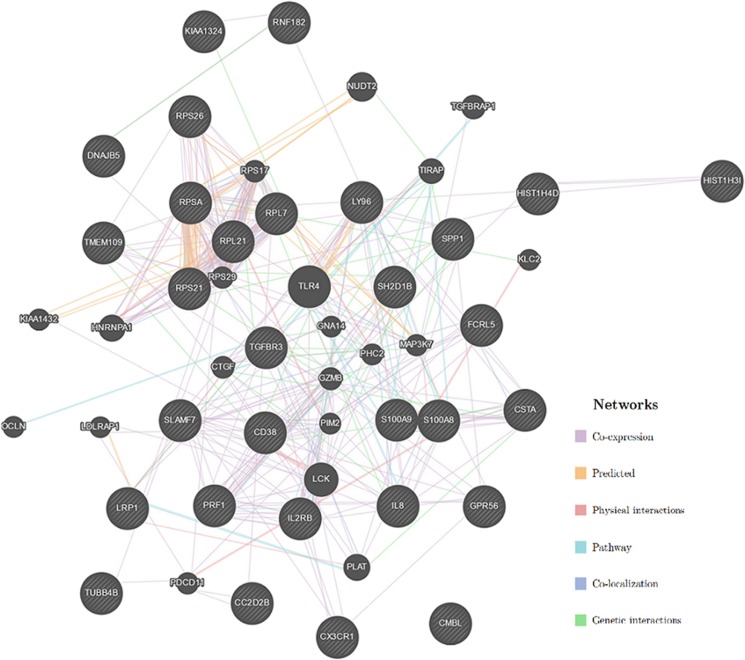
Network of genes significantly modulated and their main pathways. Gene network shows the relationships between genes from the input gene list (30 striped nodes in black) and genes strictly related from literature (small nodes in black) connected (with edges) according to the functional association networks from the databases. Different lines and colors denote the different type of interactions: in purple co-expression, in orange predicted, in blue co-localization, in green genetic interactions, in light red physical interactions, in light blue pathway.

For network analyses, we first sorted the genes that we found differentially expressed between the two groups according to the FC values, and then we chose 30 genes that resulted to be differentially modulated with a larger effect size (with the highest or lowest FC) as a query gene set for GeneMANIA tool, in order to build up a gene-network. As shown in Figure 1, all the selected genes tightly interact each other with physical interactions (26.3%) and co-expression (38.2%) among the most significant types of interactions. Several genes presented more than ten interactions, such as Perforin-1 (PRF1), S100 calcium binding protein A8 (S100A8), and interleukin-8 (IL-8). Moreover, by using Pathway Analyses Tool within GeneMania, we also found that these tightly interacting genes are mainly involved in immune and inflammation related signaling, such as the Toll-like receptor signaling, cytokine–cytokine receptor interaction, natural killer cell signaling, IL-2 signaling and chemokine receptor-chemokine interaction.

### Leukocyte telomere length in subjects exposed to SLEs

In order to study the impact of recent SLEs on peripheral blood telomere length and the possible underlying mechanisms, we investigated the relative telomere length in the genomic DNA of the same subjects exposed or not to recent SLEs, and subsequently we performed correlation analyses between telomere length and the transcriptome profile.

Telomere length was significantly reduced (by 19.1%) in subjects exposed to recent SLEs (mean of relative normalized expression±s.e.m.: 2.12±0.12 vs 2.62±0.13 in subjects exposed vs subjects not exposed to SLEs, respectively; *P*=0.03). As reported in the data on the demographic features, age was not significantly different between the two study groups and there was no significant correlation between age and leukocyte telomere length; however, as age is a well-known variable influencing the leukocyte telomere length, we also run the analyses adding age as covariate and we found that the difference in leukocyte telomere length remained significant (*P*=0.04).

Correlation analyses between leukocyte telomere length and others demographic features as well as with HAMD score, cortisol levels or smoking revealed no significant association for all the tested variables (all *P*-values >0.05).

Correlation analyses between leukocyte telomere length and transcriptomic profile identified a data set of 405 genes significantly associated with telomere length reduction (Supplementary Table 5), which resulted to be involved in 56 pathways, with natural killer cell signaling, IL-1 signaling, MIF regulation of innate immunity and IL-6 signaling as the most significant pathways (see Table 3 for the entire list of pathways modulated).

**Figure 2:**
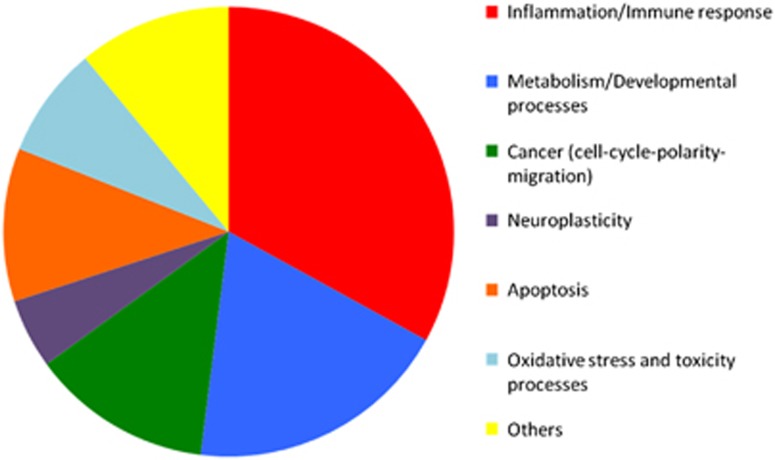
Biological processes pie chart. Pathways related both to SLEs and telomere shortening grouped according to their associated biological processes. SLE, stressful life events.

The role classification of all the 56 significant pathways has been summarized in a pie chart (see Figure 2), where the inflammatory-related processes, metabolism- and developmental-related processes, cancer and neuroplasticity, apoptosis, oxidative stress and toxicity related processes are the main represented biological processes.

## Discussion

To our knowledge, this is the first study investigating the effect of recent SLEs occurring in adulthood in peripheral blood using a whole-genome transcriptomic approach. Moreover, we have coupled the transcriptomic data with leukocyte telomere length analyses in order to study possible mechanisms underlying the effect of stress on telomere shortening.

The transcriptomic analyses revealed that 207 genes are significantly differentially expressed in subjects exposed to at least one episode of severe recent SLE as compared with not exposed subjects. Among the top genes, we found PRF1, IL-8, S100A8 and S100 calcium binding protein A9 (S100A9), together with many other genes, involved in the modulation of immune/inflammatory systems.

PRF1 is an important modulator within the cytolytic activity of both natural killer cells and cytotoxic CD8^+^ T-cells.^57^ It codes for the cytolytic protein Perforin stored in granules in T-cells and natural killer cells and it is involved in cellular defence response and programmed cell death.^58^ Indeed, mutations within *PRF1* gene and defects in its expression can cause an abnormal function of the immune system.^59^

IL-8 is a proinflammatory cytokine, member of the CXC chemokine subfamily. IL-8, also known as neutrophil chemotactic factor, induces chemotaxis and phagocytosis in target cells, primarily neutrophils, inducing them to migrate toward the site of infection.^60^ Elevated IL-8 levels have been found in patients with inflammatory-related diseases, such as Alzheimer's disease,^61^ major depressive disorder^62, 63, 64^ and cardiovascular diseases.^65, 66^

S100A8/A9 acts as a chemotactic molecule expressed by neutrophils, monocytes and macrophages.^67, 68^ In particular, S100A8/A9, released by primed myeloid cells under inflammatory conditions, promotes further leukocytes recruitment, thus enhancing a chronic inflammatory state.^69^

Taking into consideration the role of these genes, the presence of a significant reduction in PRF1 peripheral blood expression levels together with higher IL-8 and S100A8/A9 expression levels suggests an altered inflammatory response in association with SLEs exposure. This finding is supported by pathway analysis run on the 207 significantly differentially expressed genes, which revealed an enrichment in pathways related to metabolism (such as superpathway of methionine degradation, cysteine biosynthesis III, glycine biosynthesis I, TCA cycle II) and inflammation/immune responses (such as IL-17A in psoriasis, caveolar-mediated endocytosis signaling, natural killer cell signaling, T-cell receptor signaling).

Up to now, only few studies have investigated the impact of recent SLEs occurring in adulthood on immune and inflammatory mechanisms, but the findings are restricted to natural killer cells. Indeed, a meta-analysis conducted by Segerstrom and Miller (2004) shows a positive association between the number of recent SLEs and the increased number of circulating natural killer cells.^70^ Our study represents the first evidence, coming from a whole-genome transcriptomic approach, indicating that, besides stressful experiences early in life,^71, 72, 73^ also recent SLEs, experienced in adulthood, can affect the same biological systems. This suggests that recent SLEs may cause an enhanced vulnerability for the development of several illnesses, including psychiatric disorders or act as precipitating factor in already vulnerable individuals, via an activation of the inflammatory/immune system.

In the same group of subjects who experienced recent SLEs in adulthood we also reported the presence of shorter leukocyte telomere length. This finding is in line with some recent data on the topic. Verhoeven *et al.*^19^ investigated both the effects of childhood trauma and recent SLEs on leukocyte telomere length, reporting a negative association only with recent SLEs. Moreover, Parks *et al.*^74^ reported shorter leukocyte telomere length in female subjects exposed, in adulthood, to a major loss, whereas, none of the other recent major examined stressors were significantly associated with telomere length. A recent prospective study also found that recent SLEs predicted the rate of leukocyte telomere shortening over a 1-year period.^75^ However, it remains still unknown which are the specific stressors that affect telomere length, how long these effects last and importantly, whether telomere shortening could be potentially reversible.^76^

The correlation analysis between telomere length and transcriptomic data indicated 405 significantly correlated genes that belong to pathways highly related to inflammation, immune system and oxidative stress. In particular, among the top pathways associated both with SLEs exposure and leukocyte telomere length reduction, we identified the natural killer cell signaling, iNOS signaling, MIF-mediated glucocorticoid regulation and MIF regulation of innate immunity. Over the years, several studies have shown the impact of stressful life events on the blood levels and the activity of circulating natural killer cells.^70, 77^ Natural killer cells represent the first line of defence against virally infected cells, and immunological studies proposed that natural killer cells immunosenescence could contribute to the higher incidence of infections that is observed in older adults.^70, 78, 79^ Natural killer cells can also amplify immune responses by enhancing the early phases of an adaptive immune response and by promoting dendritic cell maturation and T-cell differentiation.

The inducible isoform iNOS, which produces large amount of nitric oxide (NO), has a key role in defence mechanisms. NO is synthesized by many cell types in response to cytokines activation. It is an important factor in the response against parasites, bacterial infections, and tumor growth and it has a key role in many diseases with an autoimmune etiology.^80^ In basal conditions, it has anti-inflammatory effects, but under stress conditions NO acts as a proinflammatory mediator due to its overproduction.^81^ *In vivo* evidence from Stadler and collaborators suggest a role for iNOS also in oxidative stress processes and free radicals production.^82^

MIF is a cytokine with a known ability to prevent random migration of macrophages. It is released from intracellular pools by T-lymphocytes, B-lymphocytes, monocytes, macrophages, dendritic cells, neutrophils, eosinophils, mast cells and basophils. It is widely distributed in several tissues^83^ and its release is triggered by cells exposure to microbial products, proinflammatory cytokines, or specific antigens. Upon release, it acts in an autocrine and paracrine way to induce production of proinflammatory cytokines^84^ and it also opposes the anti-inflammatory activity of glucocorticoids.^85^ It has been suggested to be involved in the pathogenesis of several immune and inflammatory disorders such as asthma, pulmonary fibrosis and rheumatoid arthritis,^86, 87^ as well as in psychiatric disorders.^88, 89^ In our previous findings MIF blood levels were higher in depressed patients as compared to controls and this alteration was associated with a poor response to conventional antidepressants.^63, 64^

Although all these pathways share a role in immune and inflammatory processes, their possible role in association with the effect of SLEs on leukocyte telomere length has not fully been elucidated yet. Indeed, although telomere shortening during lifespan is part of the physiological aging process, adverse factors such as environmental and toxic stressors can increase the rate of telomere attrition, thus leading to telomere shortening and to an acceleration of the aging process.^90, 91, 92^ Kiecolt-Glaser *et al.*^93^ reported a correlation between increased inflammatory biomarkers, like IL-6 and tumor necrosis factor-α, associated to childhood adversities exposure and accelerated leukocyte telomere shortening mechanisms. Telomere attrition due to oxidative damage, promoted by iNOS signaling modulation, is another possible cause for an increased rate of telomere shortening.^94^ The G-rich telomeric sequences are particularly sensitive to oxidative stress and the consequent damage may also affect the binding ability of the telosome/shelterin complex, that is crucial for its protective role in telomere maintenance,^95^ further leading to telomere dysfunction and cellular senescence.^96, 97^ A recent review summarizes all these results and strictly links inflammatory mechanisms with telomere shortening in aging processes, pointing out the importance of oxidative stress. Our results fit into this framework, highlighting the possible synergistic role of factors related both to inflammation and to oxidative stress in mediating peripheral telomere shortening. However, the exact role of each component and the real causality effect is still under investigation.

Limitations of our study have to be mentioned. First, we focused our analyses only on recent severe SLEs in adulthood, so we are not able to exclude that also recent mild SLEs and/or SLEs occurred in the 6 months preceding the assessment could have had an effect on the expression of the 207 genes differentially expressed in our sample. Second, we did not perform a longitudinal follow up of the subjects, so we are not able to assess whether differences in gene expression and in leukocyte telomere shortening, can predict future poor health outcomes and also, whether these differences are maintained over time. Moreover, we have analyzed the telomere length in a single cell type (whole blood cells) and just at one-time point, thus, repeated measurements at different time points and a validation in specific cell types could be useful to implement our findings.

In conclusion, our data suggest immune response, inflammation and oxidative stress as principal processes affected by SLEs; moreover, these systems could be associated with the negative effect of SLEs on leukocyte telomere length. The modulation of these mechanisms may underlie the clinical association between the exposure to recent SLEs in adulthood and an enhanced vulnerability to develop illnesses, as well as the ability of SLEs to act as precipitating factors in already vulnerable individuals. However, this hypothesis deserves further clarifications.

## Figures and Tables

**Table 1:** Top significantly modulated genes by SLEs exposure

	*Gene Assignment*	*Gene symbol*	*Fold change (SLE vs. No SLE)*
1	CD38 molecule	*CD38*	−1.4
2	Ring finger protein 182	*RNF182*	−1.4
3	SLAM family member 7	*SLAMF7*	−1.4
4	Perforin-1 (pore forming protein)	*PRF1*	−1.4
5	SH2 domain containing 1B	*SH2D1B*	−1.4
6	Immunoglobulin heavy variable 3–33	*IGHV3-33*	−1.3
7	Immunoglobulin lambda joining 3	*IGLJ3*	−1.3
8	Immunoglobulin heavy variable 3–38	*IGHV3-38*	−1.3
9	Fc receptor-like 5	*FCRL5*	−1.3
10	Histone cluster 1, H3i	*HIST1H3I*	−1.3
11	G-protein-coupled receptor 56	*GPR56*	−1.3
12	Transforming growth factor, beta receptor III	*TGFBR3*	−1.3
13	S100 calcium binding protein A9	*S100A9*	1.2
14	Cystatin A (stefin A)	*CSTA*	1.2
15	Carboxymethylenebutenolidase homolog	*CMBL*	1.2
16	Ribosomal protein S26	*RPS26*	1.2
17	RNA, 5S ribosomal 399	*RN5S399*	1.3
18	Ribosomal protein L7	*RPL7*	1.3
19	Interleukin 8	*IL8*	1.3
20	Tubulin, beta 4B class Ivb	*TUBB4B*	1.3
21	Lymphocyte antigen 96	*LY96*	1.3
22	Ribosomal protein L21	*RPL21*	1.3
23	S100 calcium binding protein A8	*S100A8*	1.4
24	KIAA1324	*KIAA1324*	1.6

Abbreviation: SLE, stressful life event.

Selection of the 24 genes with the lowest or highest values of fold change (all *q*-value<0.05), 12 downregulated and 12 upregulated.

**Table 2:** Pathways differentially modulated in subjects exposed to SLEs (*P*-value<0.05)

	*Pathway*	*Molecules*
1	Role of IL-17A in psoriasis	CXCL8, S100A9, S100A8, CXCL1
2	Caveolar-mediated endocytosis signaling	FLNB, FLNA, ABL1, ITGAL, ITGB7
3	Natural killer cell signaling	CD247, NCR1, LAT, ZAP70, KIR3DL2, SH2D1B
4	Tumoricidal function of hepatic natural killer cells	PRF1, GZMB, ITGAL
5	Virus entry via endocytic pathways	FLNB, FLNA, ABL1, ITGAL, ITGB7
6	Cytotoxic T lymphocyte-mediated apoptosis of target cells	CD247, PRF1, GZMB
7	Superpathway of methionine degradation	FTSJ1, GOT2, AHCY
8	Role of IL-17F in allergic inflammatory airway diseases	CXCL8, CCL4, CXCL1
9	CTLA4 signaling in cytotoxic T lymphocytes	CD247, PPP2R5D, LAT, ZAP70
10	Granzyme B signaling	PRF1, GZMB
11	Crosstalk between dendritic cells and natural killer cells	PRF1, KIR3DL2, ITGAL, IL2RB
12	Methionine degradation I (to Homocysteine)	FTSJ1, AHCY
13	Differential regulation of cytokine production in macrophages and T helper cells by IL-17A and IL-17F	CCL4, CXCL1
14	T-cell receptor signaling	CD247, PTPRH, LAT, ZAP70
15	Cysteine biosynthesis III (mammalia)	FTSJ1, AHCY
16	Granzyme A signaling	PRF1, HIST1H1E
17	iCOS-iCOSL signaling in T helper cells	CD247, LAT, ZAP70, IL2RB
18	Glycine biosynthesis I	SHMT2
19	Differential regulation of cytokine production in intestinal epithelial cells by IL-17A and IL-17F	CCL4, CXCL1
20	TCA cycle II (eukaryotic)	SDHA, ACO1
21	IL-17A signaling in gastric cells	CXCL8, CXCL1
22	Cell cycle control of chromosomal replication	MCM3, CDK6
23	NADH repair	APOA1BP
24	5-aminoimidazole ribonucleotide biosynthesis I	GART
25	Glutamate degradation II and aspartate biosynthesis	GOT2
26	Aspartate biosynthesis	GOT2
27	EIF2 signaling	RPS26, RPL21, RPS21, RPL7, RPSA
28	TREM1 signaling	SIGIRR, CXCL8, NLRC3
29	Cyclins and cell cycle regulation	PPP2R5D, CDK6, ABL1
30	Proline biosynthesis I	ALDH18A1
31	l-Cysteine degradation I	GOT2
32	Phenylalanine degradation I (Aerobic)	QDPR
33	Regulation of IL-2 expression in T lymphocytes	CD247, LAT, ZAP70
34	Ceramide signaling	SMPD4, S1PR5, PPP2R5D
35	Tetrahydrofolate salvage from 5,10-methenyltetrahydrofolate	GART
36	dTMP *de novo* biosynthesis	SHMT2
37	Folate polyglutamylation	SHMT2
38	Regulation of eIF4 and p70S6K signaling	RPS26, PPP2R5D, RPS21, RPSA

Abbreviations: IL, interleukin; SLE, stressful life event.

All the 38 pathways obtained from Ingenuity pathway analysis (*P*-value<0.05) using as input gene set all the 207 genes significantly modulated in subjects exposed to SLEs.

**Table 3:** Pathways significantly correlated with telomere shortening in association with SLEs exposures

	*Pathway*	*Molecules*
1	Natural killer cell signaling	KIR3DL1, RRAS2, LAIR1, INPP5B, SYK, MAPK3, KIR3DL2, KIR2DL4, KIR3DL3
2	Phosphatidylglycerol biosynthesis II	GPAM, LPCAT4, PTPMT1, AGPAT1
3	Prostate cancer signaling	RRAS2, FOXO1, PA2G4, MAPK3, NFKBIE, NFKB2, GSTP1
4	Ephrin B signaling	GNAS, RGS3, MAPK3, GNAO1, ACP1, HNRNPK
5	CDP-diacylglycerol biosynthesis I	GPAM, LPCAT4, AGPAT1
6	MIF-mediated glucocorticoid regulation	MAPK3, NFKBIE, CD14, NFKB2
7	Triacylglycerol biosynthesis	GPAM, LPCAT4, AGPAT1, PLPP1
8	Role of NFAT in regulation of the immune response	GNAS, RRAS2, SYK, MAPK3, NFKBIE, GNAO1, MS4A2, MEF2A, NFKB2
9	Crosstalk between dendritic cells and natural killer cells	KIR3DL1, FSCN1, KIR3DL2, NFKB2, KIR2DL4, KIR3DL3
10	IL-1 signaling	IL1A, GNAS, NFKBIE, GNAO1, NFKB2, IRAK2
11	MIF regulation of innate immunity	MAPK3, NFKBIE, CD14, NFKB2
12	iNOS signaling	NFKBIE, CD14, NFKB2, IRAK2
13	LPS-stimulated MAPK signaling	RRAS2, MAPK3, NFKBIE, CD14, NFKB2
14	D-myo-inositol (1,4,5)-trisphosphate biosynthesis	PIP4K2B, PI4K2B, PLCH1
15	TNFR1 signaling	NAIP, CRADD, NFKBIE, NFKB2
16	PDGF signaling	RRAS2, ABL2, INPP5B, MAPK3, ACP1
17	fMLP signaling in neutrophils	ACTR2, GNAS, RRAS2, MAPK3, NFKBIE, NFKB2
18	Ephrin receptor signaling	ACTR2, GNAS, RGS3, RRAS2, PTPN13, MAPK3, GNAO1, ACP1
19	TNFR2 signaling	NAIP, NFKBIE, NFKB2
20	Glutathione-mediated detoxification	HPGDS, ANPEP, GSTP1
21	PPARÎ±/RXRÎ± activation	GNAS, RRAS2, PRKAB1, MAPK3, NFKBIE, CYP2C18, NFKB2, ACVR1C
22	Epithelial adherens junction signaling	EPN2, ACTR2, RRAS2, LMO7, TUBB4A, ACVR1C, FARP2
23	Histamine biosynthesis	HDC
24	Alanine biosynthesis III	NFS1
25	4-1BB signaling in T lymphocytes	MAPK3, NFKBIE, NFKB2
26	IL-6 signaling	IL1A, RRAS2, MAPK3, NFKBIE, CD14, NFKB2
27	Choline biosynthesis III	PLD3, PHKA1
28	Apoptosis signaling	NAIP, RRAS2, MAPK3, NFKBIE, NFKB2
29	TWEAK signaling	NAIP, NFKBIE, NFKB2
30	PI3K/AKT signaling	RRAS2, FOXO1, INPP5B, MAPK3, NFKBIE, NFKB2
31	IL-17A signaling in fibroblasts	MAPK3, NFKBIE, NFKB2
32	PPAR signaling	IL1A, RRAS2, MAPK3, NFKBIE, NFKB2
33	Superpathway of inositol phosphate compounds	ATP1A1, PTPN13, NUDT9, INPP5B, PIP4K2B, ACP1, PI4K2B, PLCH1
34	IL-15 signaling	RRAS2, SYK, MAPK3, NFKB2
35	Role of PI3K/AKT signaling in the pathogenesis of influenza	IFNA8, MAPK3, NFKBIE, NFKB2
36	Hepatic cholestasis	IL1A, GNAS, ABCC2, NFKBIE, CD14, NFKB2, IRAK2
37	Eicosanoid signaling	PTGFR, DPEP3, ALOX5AP, HPGDS
38	Phospholipase C signaling	ARHGEF5, GNAS, PLD3, RRAS2, SYK, MAPK3, MEF2A, NFKB2, RHOH
39	Angiopoietin signaling	RRAS2, FOXO1, NFKBIE, NFKB2
40	Erythropoietin signaling	RRAS2, MAPK3, NFKBIE, NFKB2
41	Insulin receptor signaling	RRAS2, FOXO1, TRIP10, INPP5B, MAPK3, ASIC3
42	Antioxidant action of vitamin C	PLD3, MAPK3, NFKBIE, NFKB2, SLC2A3
43	IL-10 signaling	IL1A, NFKBIE, CD14, NFKB2
44	Spermine biosynthesis	SMS
45	Cardiolipin biosynthesis II	PTPMT1
46	Putrescine biosynthesis III	AZIN2
47	Cholecystokinin/gastrin-mediated signaling	IL1A, RRAS2, MAPK3, MEF2A, RHOH
48	Small cell lung cancer signaling	PA2G4, NFKBIE, NFKB2, SKP2
49	PEDF signaling	RRAS2, MAPK3, NFKBIE, NFKB2
50	B-cell receptor signaling	RRAS2, FOXO1, INPP5B, SYK, MAPK3, NFKBIE, NFKB2
51	Rac signaling	ACTR2, RRAS2, MAPK3, PIP4K2B, NFKB2
52	Role of RIG1-like receptors in antiviral innate immunity	IFNA8, NFKBIE, NFKB2
53	Systemic lupus erythematosus signaling	IL1A, RRAS2, IFNA8, PRPF3, MAPK3, SNRPB2, PRPF6, SNRPE
54	NF-KB activation by viruses	RRAS2, MAPK3, NFKBIE, NFKB2
55	Toll-like receptor signaling	IL1A, CD14, NFKB2, IRAK2
56	Fc epsilon RI signaling	RRAS2, INPP5B, SYK, MAPK3, MS4A2

Abbreviations: IL, interleukin; SLE, stressful life events. All the 56 pathways from Ingenuity pathway analysis, using as input gene set the 405 genes significantly correlated between telomere shortening and SLEs (*P*-value<0.05).
