# Supplementary material for: Transcriptomic analyses and leukocyte telomere length measurement in subjects exposed to severe recent stressful life events
Source: Transl Psychiatry. 2017 Feb 21;7(2):e1042–. doi: 10.1038/tp.2017.5 (PMC5438034; doi:10.1038/tp.2017.5)
Supplement: Supplementary Information [file tp20175x1.docx]

**Revised Manuscript ID 2016TP000571**

**Supplementary material**

**Supplementary table 1. Demographic and clinical features of the study groups**

|  | **Gender (% females)** | **Age (mean±SD)** | **BMI (mean±SD)** | **Smoking (%)** | **HAMD (mean±SD)** | **Ethnicity (% Caucasians)** |
| --- | --- | --- | --- | --- | --- | --- |
| SLE (n=21) | 48 | 37.33**±**12.98 | 24.08**±**4.30 | 33 | 2.19**±**2.66 | 100 |
| No SLE (n=32) | 58 | 35.77**±**11.74 | 23.54**±**4.29 | 26 | 2.00**±**2.31 | 97 |
| p-value | 0.47 | 0.65 | 0.65 | 0.57 | 0.78 | 0.42 |

**Supplementary Table 2. List of genes significantly differentially expressed in subjects exposed to SLEs as compared to not exposed subjects (q-value<0.05)**

|  | **Gene assignment** | **Gene symbol** | **FC** |
| --- | --- | --- | --- |
| 1 | CD38 molecule | CD38 | -1.4 |
| 2 | ring finger protein 182 | RNF182 | -1.4 |
| 3 | SLAM family member 7 | SLAMF7 | -1.4 |
| 4 | perforin 1 (pore forming protein) | PRF1 | -1.4 |
| 5 | SH2 domain containing 1B | SH2D1B | -1.4 |
| 6 | immunoglobulin heavy variable 3-33 | IGHV3-33 | -1.3 |
| 7 | immunoglobulin lambda joining 3 | IGLJ3 | -1.3 |
| 8 | immunoglobulin heavy variable 3-38 | IGHV3-38 | -1.3 |
| 9 | Fc receptor-like 5 | FCRL5 | -1.3 |
| 10 | histone cluster 1, H3i | HIST1H3I | -1.3 |
| 11 | G protein-coupled receptor 56 | GPR56 | -1.3 |
| 12 | transforming growth factor, beta receptor III | TGFBR3 | -1.3 |
| 13 | histone cluster 1, H4d | HIST1H4D | -1.3 |
| 14 | immunoglobulin heavy variable 3-74 | IGHV3-74 | -1.3 |
| 15 | transmembrane protein 109 | TMEM109 | -1.3 |
| 16 | interleukin 2 receptor, beta | IL2RB | -1.3 |
| 17 | chemokine (C-X3-C motif) receptor 1 | CX3CR1 | -1.2 |
| 18 | low density lipoprotein receptor-related protein 1 | LRP1 | -1.2 |
| 19 | ribosomal protein SA | RPSA | -1.2 |
| 20 | immunoglobulin heavy variable 3-72 | IGHV3-72 | -1.2 |
| 21 | cystatin F (leukocystatin) | CST7 | -1.2 |
| 22 | natural killer cell group 7 sequence | NKG7 | -1.2 |
| 23 | spondin 2, extracellular matrix protein | SPON2 | -1.2 |
| 24 | CD247 molecule | CD247 | -1.2 |
| 25 | coagulation factor II (thrombin) receptor | F2R | -1.2 |
| 26 | natural cytotoxicity triggering receptor 1 | NCR1 | -1.2 |
| 27 | histone cluster 1, H1e | HIST1H1E | -1.2 |
| 28 | killer cell immunoglobulin-like receptor | KIR3DL2 | -1.2 |
| 29 | transmembrane protein 229B | TMEM229B | -1.2 |
| 30 | neuromedin U receptor 1 | NMUR1 | -1.2 |
| 31 | hypoxia up-regulated 1 | HYOU1 | -1.2 |
| 32 | cyclin-dependent kinase 6 | CDK6 | -1.2 |
| 33 | histone cluster 1, H2ab | HIST1H2AB | -1.2 |
| 34 | zinc finger protein 749 | ZNF749 | -1.2 |
| 35 | patched 1 | PTCH1 | -1.2 |
| 36 | lymphocyte transmembrane adaptor 1 | LAX1 | -1.2 |
| 37 | zeta-chain (TCR) associated protein kinase 70kDa | ZAP70 | -1.2 |
| 38 | SH2 domain containing 2A | SH2D2A | -1.2 |
| 39 | GTPase, IMAP family member 6 | GIMAP6 | -1.2 |
| 40 | minichromosome maintenance complex component 3 | MCM3 | -1.2 |
| 41 | tetratricopeptide repeat domain 38 | TTC38 | -1.2 |
| 42 | T-box 21 | TBX21 | -1.2 |
| 43 | chromosome 16 open reading frame 80 | C16orf80 | -1.2 |
| 44 | B-cell receptor-associated protein 31 | BCAP31 | -1.2 |
| 45 | ATP-binding cassette, sub-family A (ABC1), member 2 | ABCA2 | -1.2 |
| 46 | glucosidase, alpha; neutral AB | GANAB | -1.2 |
| 47 | X-box binding protein 1 | XBP1 | -1.2 |
| 48 | ARP1 actin-related protein 1 homolog B, centractin beta | ACTR1B | -1.2 |
| 49 | granzyme B | GZMB | -1.2 |
| 50 | chromosome 15 open reading frame 38 | C15orf38 | -1.2 |
| 51 | 1-aminocyclopropane-1-carboxylate synthase homolog | ACCS | -1.2 |
| 52 | integrin, alpha L | ITGAL | -1.2 |
| 53 | sema domain, immunoglobulin domain (Ig) | SEMA4C | -1.2 |
| 54 | atlastin GTPase 3 | ATL3 | -1.2 |
| 55 | filamin A, alpha | FLNA | -1.2 |
| 56 | dehydrogenase/reductase (SDR family) member 3 | DHRS3 | -1.2 |
| 57 | chemokine (C-C motif) ligand 4 | CCL4 | -1.2 |
| 58 | synaptotagmin XI | SYT11 | -1.2 |
| 59 | metaxin 3 | MTX3 | -1.2 |
| 60 | ADP-ribosylation factor-like 4C | ARL4C | -1.2 |
| 61 | RuvB-like 1 (E. coli) | RUVBL1 | -1.2 |
| 62 | glutamic-oxaloacetic transaminase 2, mitochondrial | GOT2 | -1.2 |
| 63 | metastasis associated 1 family, member 2 | MTA2 | -1.2 |
| 64 | tribbles homolog 2 (Drosophila) | TRIB2 | -1.2 |
| 65 | runt-related transcription factor 3 | RUNX3 | -1.2 |
| 66 | GCN1 general control of amino-acid synthesis 1-like 1 | GCN1L1 | -1.2 |
| 67 | zinc finger and BTB domain containing 16 | ZBTB16 | -1.1 |
| 68 | ubiquitin-conjugating enzyme E2G 2 | UBE2G2 | -1.1 |
| 69 | apolipoprotein B mRNA editing enzyme | APOBEC3G | -1.1 |
| 70 | coenzyme Q10 homolog A (S. cerevisiae) | COQ10A | -1.1 |
| 71 | CNDP dipeptidase 2 (metallopeptidase M20 family) | CNDP2 | -1.1 |
| 72 | leucine-zipper-like transcription regulator 1 | LZTR1 | -1.1 |
| 73 | poly(A) binding protein, cytoplasmic 4 | PABPC4 | -1.1 |
| 74 | interleukin 27 receptor, alpha | IL27RA | -1.1 |
| 75 | serine hydroxymethyltransferase 2 | SHMT2 | -1.1 |
| 76 | aldehyde dehydrogenase 18 family, member A1 | ALDH18A1 | -1.1 |
| 77 | polymerase (RNA) II (DNA directed) polypeptide G | POLR2G | -1.1 |
| 78 | dolichyl-diphosphooligosaccharide--protein glycosyltransferase | DDOST | -1.1 |
| 79 | PRP19/PSO4 pre-mRNA processing factor 19 homolog | PRPF19 | -1.1 |
| 80 | polymerase (RNA) I polypeptide A, 194kDa | POLR1A | -1.1 |
| 81 | phosphoribosylglycinamide formyltransferase | GART | -1.1 |
| 82 | Mov10, Moloney leukemia virus 10, homolog | MOV10 | -1.1 |
| 83 | N-acylaminoacyl-peptide hydrolase | APEH | -1.1 |
| 84 | fucosyltransferase 11 (alpha (1,3) fucosyltransferase) | FUT11 | -1.1 |
| 85 | sphingomyelin phosphodiesterase 4, neutral membrane | SMPD4 | -1.1 |
| 86 | PRP4 pre-mRNA processing factor 4 homolog (yeast) | PRPF4 | -1.1 |
| 87 | nucleolar complex associated 2 homolog (S. cerevisiae) | NOC2L | -1.1 |
| 88 | Sad1 and UNC84 domain containing 2 | SUN2 | -1.1 |
| 89 | potassium voltage-gated channel, KQT-like subfamily, member 5 | KCNQ5 | -1.1 |
| 90 | PAN2 poly(A) specific ribonuclease subunit homolog | PAN2 | -1.1 |
| 91 | ER membrane protein complex subunit 1 | EMC1 | -1.1 |
| 92 | non-SMC element 1 homolog (S. cerevisiae) | NSMCE1 | -1.1 |
| 93 | aconitase 1, soluble | ACO1 | -1.1 |
| 94 | NOP2 nucleolar protein homolog | NOP2 | -1.1 |
| 95 | gem (nuclear organelle) associated protein 4 | GEMIN4 | -1.1 |
| 96 | intermediate filament family orphan 2 | IFFO2 | -1.1 |
| 97 | single immunoglobulin and toll-interleukin 1 receptor (TIR) | SIGIRR | -1.1 |
| 98 | integrin, beta 7 | ITGB7 | -1.1 |
| 99 | zinc finger protein 276 | ZNF276 | -1.1 |
| 100 | CD81 molecule | CD81 | -1.1 |
| 101 | AHNAK nucleoprotein | AHNAK | -1.1 |
| 102 | Rho GTPase activating protein 42 | ARHGAP42 | -1.1 |
| 103 | peroxisomal biogenesis factor 5 | PEX5 | -1.1 |
| 104 | succinate dehydrogenase complex, subunit A, flavoprotein (Fp) | SDHA | -1.1 |
| 105 | filamin B, beta | FLNB | -1.1 |
| 106 | myotubularin related protein 8 | MTMR8 | -1.1 |
| 107 | protein phosphatase 2, regulatory subunit B', delta | PPP2R5D | -1.1 |
| 108 | damage-specific DNA binding protein 2, 48kDa | DDB2 | -1.1 |
| 109 | echinoderm microtubule associated protein like 3 | EML3 | -1.1 |
| 110 | quinoid dihydropteridine reductase | QDPR | -1.1 |
| 111 | growth factor independent 1 transcription repressor | GFI1 | -1.1 |
| 112 | asparagine-linked glycosylation 10, alpha-1,2-glucosyltransferase | ALG10 | -1.1 |
| 113 | DEAD (Asp-Glu-Ala-Asp) box helicase 56 | DDX56 | -1.1 |
| 114 | UbiA prenyltransferase domain containing 1 | UBIAD1 | -1.1 |
| 115 | apolipoprotein A-I binding protein | APOA1BP | -1.1 |
| 116 | emopamil binding protein (sterol isomerase) | EBP | -1.1 |
| 117 | megalencephalic leukoencephalopathy with subcortical cysts 1 | MLC1 | -1.1 |
| 118 | RAB7, member RAS oncogene family-like 1 | RAB7L1 | -1.1 |
| 119 | histone cluster 1, H2ak | HIST1H2AK | -1.1 |
| 120 | Kruppel-like factor 8 | KLF8 | -1.1 |
| 121 | leucine rich repeat containing 59 | LRRC59 | -1.1 |
| 122 | zinc finger, DHHC-type containing 9 | ZDHHC9 | -1.1 |
| 123 | gem (nuclear organelle) associated protein 5 | GEMIN5 | -1.1 |
| 124 | retinol saturase (all-trans-retinol 13,14-reductase) | RETSAT | -1.1 |
| 125 | chromosome X open reading frame 40B | CXorf40B | -1.1 |
| 126 | CXXC finger protein 5 | CXXC5 | -1.1 |
| 127 | WD repeat domain 46 | WDR46 | -1.1 |
| 128 | porcupine homolog (Drosophila) | PORCN | -1.1 |
| 129 | Kruppel-like factor 10 | KLF10 | -1.1 |
| 130 | mitochondrial ribosomal protein S18B | MRPS18B | -1.1 |
| 131 | chromosome 3 open reading frame 37 | C3orf37 | -1.1 |
| 132 | leucine zipper, down-regulated in cancer 1-like | LDOC1L | -1.1 |
| 133 | legumain | LGMN | -1.1 |
| 134 | ectonucleoside triphosphate diphosphohydrolase 7 | ENTPD7 | -1.1 |
| 135 | mex-3 homolog C (C. elegans) | MEX3C | -1.1 |
| 136 | septin 1 | SEPT1 | -1.1 |
| 137 | NudC domain containing 3 | NUDCD3 | -1.1 |
| 138 | UDP-N-acteylglucosamine pyrophosphorylase 1 | UAP1 | -1.1 |
| 139 | exostosin 2 | EXT2 | -1.1 |
| 140 | coiled-coil domain containing 101 | CCDC101 | -1.1 |
| 141 | enhancer of mRNA decapping 4 | EDC4 | -1.1 |
| 142 | solute carrier family 35, member C2 | SLC35C2 | -1.1 |
| 143 | small glutamine-rich tetratricopeptide repeat (TPR) | SGTA | -1.1 |
| 144 | ATP-binding cassette, sub-family C (CFTR/MRP), member 10 | ABCC10 | -1.1 |
| 145 | prenylcysteine oxidase 1 like | PCYOX1L | -1.1 |
| 146 | pleckstrin homology domain containing | PLEKHF1 | -1.1 |
| 147 | guanylate cyclase 1, soluble, alpha 3 | GUCY1A3 | -1.1 |
| 148 | FtsJ RNA methyltransferase homolog 1 (E. coli) | FTSJ1 | -1.1 |
| 149 | NLR family, CARD domain containing 3 | NLRC3 | -1.1 |
| 150 | aspartate beta-hydroxylase domain containing 2 | ASPHD2 | -1.1 |
| 151 | additional sex combs like 1 (Drosophila) | ASXL1 | -1.1 |
| 152 | glycerol-3-phosphate acyltransferase, mitochondrial | GPAM | -1.1 |
| 153 | mannosyl-oligosaccharide glucosidase | MOGS | -1.1 |
| 154 | methionyl-tRNA synthetase 2, mitochondrial | MARS2 | -1.1 |
| 155 | procollagen-lysine, 2-oxoglutarate 5-dioxygenase 3 | PLOD3 | -1.1 |
| 156 | HECT and RLD domain containing E3 ubiquitin protein ligase 2 | HERC2 | -1.1 |
| 157 | family with sequence similarity 156, member A | FAM156A | -1.1 |
| 158 | transmembrane protein 120B | TMEM120B | -1.1 |
| 159 | uncharacterized FLJ39653 | FLJ39653 | -1.1 |
| 160 | latent transforming growth factor beta binding protein 3 | LTBP3 | -1.1 |
| 161 | sphingosine-1-phosphate receptor 5 | S1PR5 | -1.1 |
| 162 | histone cluster 1, H2ag | HIST1H2AG | -1.1 |
| 163 | homeobox B2 | HOXB2 | -1.1 |
| 164 | protease, serine, 23 | PRSS23 | -1.1 |
| 165 | valyl-tRNA synthetase 2, mitochondrial (putative) | VARS2 | -1.1 |
| 166 | linker for activation of T cells | LAT | -1.1 |
| 167 | Enah/Vasp-like | EVL | -1.1 |
| 168 | F-box protein 31 | FBXO31 | -1.1 |
| 169 | megakaryocyte-associated tyrosine kinase | MATK | -1.1 |
| 170 | endo-beta-N-acetylglucosaminidase | ENGASE | -1.1 |
| 171 | c-abl oncogene 1, non-receptor tyrosine kinase | ABL1 | -1.1 |
| 172 | GTP binding protein 6 (putative) | GTPBP6 | -1.1 |
| 173 | CXXC finger protein 1 | CXXC1 | -1.1 |
| 174 | adenosylhomocysteinase | AHCY | -1.1 |
| 175 | plectin | PLEC | -1.1 |
| 176 | interleukin 21 receptor | IL21R | -1.1 |
| 177 | uncharacterized MGC24103 | MGC24103 | -1.1 |
| 178 | dipeptidyl-peptidase 7 | DPP7 | -1.1 |
| 179 | solute carrier family 35, member B2 | SLC35B2 | -1.1 |
| 180 | NADH dehydrogenase (ubiquinone) 1 alpha subcomplex, 8, 19kDa | NDUFA8 | -1.1 |
| 181 | ADP-ribosylation factor-like 6 interacting protein 1 | ARL6IP1 | -1.1 |
| 182 | testis expressed 34 | TEX34 | 1.1 |
| 183 | zinc finger protein 69 | ZNF69 | 1.1 |
| 184 | activating transcription factor 5 | ATF5 | 1.1 |
| 185 | transmembrane channel-like 4 | TMC4 | 1.1 |
| 186 | chromosome 8 open reading frame 12 | C8orf12 | 1.1 |
| 187 | lysozyme-like 1 | LYZL1 | 1.1 |
| 188 | protein tyrosine phosphatase, receptor type, H | PTPRH | 1.1 |
| 189 | chemokine (C-X-C motif) ligand 1 | CXCL1 | 1.1 |
| 190 | FGF-2 activity-associated protein 2 | GAFA2 | 1.1 |
| 191 | olfactory receptor, family 6, subfamily F, member 1 | OR6F1 | 1.1 |
| 192 | ribosomal protein S21 | RPS21 | 1.2 |
| 193 | secreted phosphoprotein 1 | SPP1 | 1.2 |
| 194 | coiled-coil and C2 domain containing 2B | CC2D2B | 1.2 |
| 195 | DnaJ (Hsp40) homolog, subfamily B, member 5 | DNAJB5 | 1.2 |
| 196 | S100 calcium binding protein A9 | S100A9 | 1.2 |
| 197 | cystatin A (stefin A) | CSTA | 1.2 |
| 198 | carboxymethylenebutenolidase homolog (Pseudomonas) | CMBL | 1.2 |
| 199 | ribosomal protein S26 | RPS26 | 1.2 |
| 200 | RNA, 5S ribosomal 399 | RN5S399 | 1.3 |
| 201 | ribosomal protein L7 | RPL7 | 1.3 |
| 202 | interleukin 8 | IL8 | 1.3 |
| 203 | tubulin, beta 4B class Ivb | TUBB4B | 1.3 |
| 204 | lymphocyte antigen 96 | LY96 | 1.3 |
| 205 | ribosomal protein L21 | RPL21 | 1.3 |
| 206 | S100 calcium binding protein A8 | S100A8 | 1.4 |
| 207 | KIAA1324 | KIAA1324 | 1.6 |

**Supplementary Table 3. List of genes significantly correlated with HAMD score (p-value<0.05)**

|  | **Gene assignment** | | **Gene Symbol** | | **p-value** | |
| --- | --- | --- | --- | --- | --- | --- |
| 1 | | period circadian clock 1 | | PER1 | | 0.0004 |
| 2 | | transmembrane protein 144 | | TMEM144 | | 0.0026 |
| 3 | | calpain 5 | | CAPN5 | | 0.0034 |
| 4 | | CDC42 binding protein kinase beta (DMPK-like) | | CDC42BPB | | 0.0043 |
| 5 | | ST6 (alpha-N-acetyl-neuraminyl-2,3-beta-galactosyl-1,3) | | ST6GALNAC4 | | 0.0044 |
| 6 | | sulfotransferase family, cytosolic, 1A, phenol-preferring | | SULT1A2 | | 0.0050 |
| 7 | | nuclear factor, erythroid 2-like 3 | | NFE2L3 | | 0.0051 |
| 8 | | cytochrome P450, family 4, subfamily F, polypeptide 35 | | CYP4F35P | | 0.0051 |
| 9 | | frizzled class receptor 6 | | FZD6 | | 0.0056 |
| 10 | | H1 histone family, member 0 | | H1F0 | | 0.0057 |
| 11 | | transcription factor 3 | | TCF3 | | 0.0064 |
| 12 | | period circadian clock 2 | | PER2 | | 0.0072 |
| 13 | | feline leukemia virus subgroup C cellular receptor family | | FLVCR2 | | 0.0077 |
| 14 | | nidogen 1 | | NID1 | | 0.0079 |
| 15 | | NADH dehydrogenase (ubiquinone) complex I, assembly factor 3 | | NDUFAF3 | | 0.0081 |
| 16 | | protein tyrosine phosphatase, receptor type, M | | PTPRM | | 0.0085 |
| 17 | | retinol dehydrogenase 10 (all-trans) | | RDH10 | | 0.0086 |
| 18 | | ATP5S-like | | ATP5SL | | 0.0088 |
| 19 | | ring finger protein 187 | | RNF187 | | 0.0089 |
| 20 | | transmembrane and tetratricopeptide repeat containing 1 | | TMTC1 | | 0.0092 |
| 21 | | proline rich 5 (renal) | | PRR5 | | 0.0092 |
| 22 | | solute carrier family 25 | | SLC25A19 | | 0.0093 |
| 23 | | shisa family member 7 | | SHISA7 | | 0.0095 |
| 24 | | ILF3 antisense RNA 1 (head to head) | | ILF3-AS1 | | 0.0099 |
| 25 | | serine/arginine repetitive matrix 1 | | SRRM1 | | 0.0108 |
| 26 | | plexin domain containing 1 | | PLXDC1 | | 0.0123 |
| 27 | | glucose 6 phosphatase, catalytic, 3 | | G6PC3 | | 0.0133 |
| 28 | | prostate tumor overexpressed 1 | | PTOV1 | | 0.0135 |
| 29 | | syntaxin binding protein 1 | | STXBP1 | | 0.0137 |
| 30 | | solute carrier family 6 (neurotransmitter transporter) | | SLC6A10PB | | 0.0139 |
| 31 | | lin-7 homolog B (C. elegans) | | LIN7B | | 0.0142 |
| 32 | | occludin/ELL domain containing 1 | | OCEL1 | | 0.0142 |
| 33 | | myosin IC | | MYO1C | | 0.0147 |
| 34 | | FIC domain containing | | FICD | | 0.0150 |
| 35 | | spectrin, beta, erythrocytic | | SPTB | | 0.0151 |
| 36 | | sulfotransferase family, cytosolic, 1A, phenol-preferring | | SULT1A1 | | 0.0151 |
| 37 | | coiled-coil domain containing 124 | | CCDC124 | | 0.0152 |
| 38 | | cerebellar degeneration-related protein 1, 34kDa | | CDR1 | | 0.0152 |
| 39 | | SH3 domain and tetratricopeptide repeats 1 | | SH3TC1 | | 0.0153 |
| 40 | | phosphatidic acid phosphatase type 2A | | PPAP2A | | 0.0155 |
| 41 | | KIAA0408 | | KIAA0408 | | 0.0163 |
| 42 | | D site of albumin promoter (albumin D-box) binding protein | | DBP | | 0.0164 |
| 43 | | transcobalamin II | | TCN2 | | 0.0167 |
| 44 | | DEP domain containing MTOR-interacting protein | | DEPTOR | | 0.0170 |
| 45 | | zinc finger and BTB domain containing 5 | | ZBTB5 | | 0.0171 |
| 46 | | CASP2 and RIPK1 domain containing adaptor with death domain | | CRADD | | 0.0174 |
| 47 | | interferon, alpha 2 | | IFNA2 | | 0.0182 |
| 48 | | nuclear factor of kappa light polypeptide gene enhancer | | NFKBIB | | 0.0185 |
| 49 | | PRA1 domain family, member 2 | | PRAF2 | | 0.0193 |
| 50 | | mucolipin 3 | | MCOLN3 | | 0.0193 |
| 51 | | IKAROS family zinc finger 4 (Eos) | | IKZF4 | | 0.0199 |
| 52 | | insulin-like growth factor binding protein 7 | | IGFBP7 | | 0.0201 |
| 53 | | family with sequence similarity 19 (chemokine (C-C motif)-like) | | FAM19A2 | | 0.0205 |
| 54 | | charged multivesicular body protein 2A | | CHMP2A | | 0.0207 |
| 55 | | eyes shut homolog (Drosophila) | | EYS | | 0.0208 |
| 56 | | dynein, cytoplasmic 2, heavy chain 1 | | DYNC2H1 | | 0.0210 |
| 57 | | tweety family member 3 | | TTYH3 | | 0.0212 |
| 58 | | zinc finger, DHHC-type containing 3 | | ZDHHC3 | | 0.0214 |
| 59 | | GTP cyclohydrolase I feedback regulator | | GCHFR | | 0.0218 |
| 60 | | succinate dehydrogenase complex, subunit D, integral membrane | | SDHD | | 0.0219 |
| 61 | | protein kinase C, zeta | | PRKCZ | | 0.0219 |
| 62 | | UTP14, U3 small nucleolar ribonucleoprotein, homolog A | | UTP14A | | 0.0222 |
| 63 | | pro-melanin-concentrating hormone | | PMCH | | 0.0222 |
| 64 | | F-box and leucine-rich repeat protein 12 | | FBXL12 | | 0.0226 |
| 65 | | transmembrane protein 17 | | TMEM17 | | 0.0228 |
| 66 | | ZBTB11 antisense RNA 1 | | ZBTB11-AS1 | | 0.0229 |
| 67 | | PTPRF interacting protein, binding protein 2 (liprin beta 2) | | PPFIBP2 | | 0.0229 |
| 68 | | phospholamban | | PLN | | 0.0235 |
| 69 | | solute carrier family 3 (amino acid transporter heavy chain) | | SLC3A2 | | 0.0236 |
| 70 | | cytochrome c oxidase subunit Vb | | COX5B | | 0.0238 |
| 71 | | Treacher Collins-Franceschetti syndrome 1 | | TCOF1 | | 0.0240 |
| 72 | | carnitine O-acetyltransferase | | CRAT | | 0.0244 |
| 73 | | processing of precursor 4, ribonuclease P/MRP subunit | | POP4 | | 0.0249 |
| 74 | | purinergic receptor P2X, ligand-gated ion channel, 7 | | P2RX7 | | 0.0250 |
| 75 | | zinc finger and BTB domain containing 16 | | ZBTB16 | | 0.0251 |
| 76 | | B-cell CLL/lymphoma 7A | | BCL7A | | 0.0251 |
| 77 | | chromosome 16 open reading frame 87 | | C16orf87 | | 0.0252 |
| 78 | | neurolysin (metallopeptidase M3 family) | | NLN | | 0.0252 |
| 79 | | UPF3 regulator of nonsense transcripts homolog B (yeast) | | UPF3B | | 0.0254 |
| 80 | | caspase recruitment domain family, member 17 | | CARD17 | | 0.0259 |
| 81 | | NADH dehydrogenase (ubiquinone) complex I, assembly factor 2 | | NDUFAF2 | | 0.0264 |
| 82 | | major histocompatibility complex, class II, DR beta 3 | | HLA-DRB3 | | 0.0265 |
| 83 | | CD300c molecule | | CD300C | | 0.0274 |
| 84 | | KIAA1598 | | KIAA1598 | | 0.0278 |
| 85 | | epithelial mitogen | | EPGN | | 0.0278 |
| 86 | | coiled-coil domain containing 159 | | CCDC159 | | 0.0278 |
| 87 | | apoptogenic 1, mitochondrial | | APOPT1 | | 0.0279 |
| 88 | | hippocalcin like 4 | | HPCAL4 | | 0.0282 |
| 89 | | Rho GTPase activating protein 29 | | ARHGAP29 | | 0.0283 |
| 90 | | taxilin beta | | TXLNB | | 0.0286 |
| 91 | | zinc finger protein 773 | | ZNF773 | | 0.0288 |
| 92 | | WD repeat domain 74 | | WDR74 | | 0.0289 |
| 93 | | dual specificity phosphatase 16 | | DUSP16 | | 0.0296 |
| 94 | | DEAD (Asp-Glu-Ala-Asp) box polypeptide 41 | | DDX41 | | 0.0298 |
| 95 | | sphingomyelin synthase 2 | | SGMS2 | | 0.0303 |
| 96 | | histone deacetylase 9 | | HDAC9 | | 0.0303 |
| 97 | | chromosome 11 open reading frame 49 | | C11orf49 | | 0.0306 |
| 98 | | PAX8 antisense RNA 1 | | PAX8-AS1 | | 0.0307 |
| 99 | | ribonuclease P/MRP 38kDa subunit | | RPP38 | | 0.0309 |
| 100 | | solute carrier family 25 (mitochondrial iron transporter) | | SLC25A28 | | 0.0310 |
| 101 | | UBA-like domain containing 2 | | UBALD2 | | 0.0311 |
| 102 | | Fas apoptotic inhibitory molecule | | FAIM | | 0.0314 |
| 103 | | coiled-coil domain containing 122 | | CCDC122 | | 0.0315 |
| 104 | | MPN domain containing | | MPND | | 0.0315 |
| 105 | | hexokinase 1 | | HK1 | | 0.0318 |
| 106 | | lumican | | LUM | | 0.0319 |
| 107 | | Yae1 domain containing 1 | | YAE1D1 | | 0.0319 |
| 108 | | small nuclear ribonucleoprotein polypeptide E | | SNRPE | | 0.0321 |
| 109 | | cofilin 1 (non-muscle) | | CFL1 | | 0.0321 |
| 110 | | excision repair cross-complementation group 6-like | | ERCC6L | | 0.0323 |
| 111 | | basic, immunoglobulin-like variable motif containing | | BIVM | | 0.0324 |
| 112 | | RNA binding motif protein, X-linked 2 | | RBMX2 | | 0.0328 |
| 113 | | paraneoplastic Ma antigen family-like 1 | | PNMAL1 | | 0.0328 |
| 114 | | chromosome 10 open reading frame 131 | | C10orf131 | | 0.0328 |
| 115 | | pleckstrin homology domain containing, family B (evectins) | | PLEKHB1 | | 0.0328 |
| 116 | | chromatin assembly factor 1, subunit B (p60) | | CHAF1B | | 0.0330 |
| 117 | | chimerin 2 | | CHN2 | | 0.0330 |
| 118 | | neuropilin (NRP) and tolloid (TLL)-like 1 | | NETO1 | | 0.0331 |
| 119 | | modulator of apoptosis 1 | | MOAP1 | | 0.0336 |
| 120 | | MICAL C-terminal like | | MICALCL | | 0.0341 |
| 121 | | chromosome alignment maintaining phosphoprotein 1 | | CHAMP1 | | 0.0341 |
| 122 | | profilin 1 | | PFN1 | | 0.0342 |
| 123 | | activin A receptor, type IC | | ACVR1C | | 0.0345 |
| 124 | | family with sequence similarity 184, member A | | FAM184A | | 0.0346 |
| 125 | | peptidyl-tRNA hydrolase 2 | | PTRH2 | | 0.0346 |
| 126 | | histone cluster 1, H4k | | HIST1H4K | | 0.0346 |
| 127 | | chromosome 20 open reading frame 203 | | C20orf203 | | 0.0353 |
| 128 | | ZFP69 zinc finger protein B | | ZFP69B | | 0.0353 |
| 129 | | H2.0-like homeobox | | HLX | | 0.0360 |
| 130 | | tumor necrosis factor, alpha-induced protein 6 | | TNFAIP6 | | 0.0364 |
| 131 | | testis expressed 26 | | TEX26 | | 0.0364 |
| 132 | | polymerase (DNA directed) nu | | POLN | | 0.0367 |
| 133 | | mitochondrial ribosomal protein S2 | | MRPS2 | | 0.0367 |
| 134 | | collagen-like tail subunit (single strand of homotrimer) | | COLQ | | 0.0369 |
| 135 | | lipocalin 2 | | LCN2 | | 0.0371 |
| 136 | | zinc finger, HIT-type containing 1 | | ZNHIT1 | | 0.0371 |
| 137 | | eukaryotic translation initiation factor 2 | | EIF2S2P3 | | 0.0372 |
| 138 | | ZFP28 zinc finger protein | | ZFP28 | | 0.0383 |
| 139 | | DEAD (Asp-Glu-Ala-Asp) box polypeptide 43 | | DDX43 | | 0.0384 |
| 140 | | chromosome 9 open reading frame 131 | | C9orf131 | | 0.0386 |
| 141 | | serine peptidase inhibitor, Kazal type 1 | | SPINK1 | | 0.0386 |
| 142 | | glutathione peroxidase 2 (gastrointestinal) | | GPX2 | | 0.0387 |
| 143 | | WEE1 G2 checkpoint kinase | | WEE1 | | 0.0395 |
| 144 | | crystallin, zeta (quinone reductase) | | CRYZ | | 0.0396 |
| 145 | | glutamate receptor, ionotropic, N-methyl D-aspartate-associated | | GRINA | | 0.0397 |
| 146 | | zinc finger protein 470 | | ZNF470 | | 0.0401 |
| 147 | | zinc finger homeobox 3 | | ZFHX3 | | 0.0405 |
| 148 | | zinc finger protein 213 | | ZNF213 | | 0.0405 |
| 149 | | RAS (RAD and GEM)-like GTP binding 2 | | REM2 | | 0.0405 |
| 150 | | polymerase (RNA) I polypeptide A, 194kDa | | POLR1A | | 0.0408 |
| 151 | | small nuclear ribonucleoprotein polypeptide N | | SNRPN | | 0.0413 |
| 152 | | glia maturation factor, gamma | | GMFG | | 0.0420 |
| 153 | | YY1 transcription factor | | YY1 | | 0.0421 |
| 154 | | phosphatidylethanolamine N-methyltransferase | | PEMT | | 0.0423 |
| 155 | | MAP7 domain containing 3 | | MAP7D3 | | 0.0424 |
| 156 | | KIAA0825 | | KIAA0825 | | 0.0425 |
| 157 | | Sp2 transcription factor | | SP2 | | 0.0425 |
| 158 | | serpin peptidase inhibitor, clade B (ovalbumin), member 9 | | SERPINB9P1 | | 0.0426 |
| 159 | | zinc finger protein 658 | | ZNF658 | | 0.0427 |
| 160 | | ceroid-lipofuscinosis, neuronal 8 | | CLN8 | | 0.0429 |
| 161 | | myeloid/lymphoid or mixed-lineage leukemia | | MLLT4 | | 0.0430 |
| 162 | | fibulin 2 | | FBLN2 | | 0.0431 |
| 163 | | M-phase phosphoprotein 10 | | MPHOSPH10 | | 0.0431 |
| 164 | | arylsulfatase B | | ARSB | | 0.0433 |
| 165 | | lanosterol synthase (2,3-oxidosqualene-lanosterol cyclase) | | LSS | | 0.0436 |
| 166 | | G protein-coupled receptor 125 | | GPR125 | | 0.0439 |
| 167 | | cytochrome P450, family 4, subfamily F, polypeptide 11 | | CYP4F11 | | 0.0439 |
| 168 | | chromosome 9 open reading frame 89 | | C9orf89 | | 0.0440 |
| 169 | | cleavage stimulation factor, 3 pre-RNA, subunit 2, 64kDa | | CSTF2 | | 0.0441 |
| 170 | | leucine rich repeat containing 8 family, member A | | LRRC8A | | 0.0442 |
| 171 | | SHANK-associated RH domain interactor | | SHARPIN | | 0.0447 |
| 172 | | G protein-coupled receptor 124 | | GPR124 | | 0.0448 |
| 173 | | filamin A interacting protein 1-like | | FILIP1L | | 0.0450 |
| 174 | | solute carrier family 9, subfamily A | | SLC9A7 | | 0.0453 |
| 175 | | cystatin A (stefin A) | | CSTA | | 0.0453 |
| 176 | | RNA pseudouridylate synthase domain containing 3 | | RPUSD3 | | 0.0456 |
| 177 | | nucleosome assembly protein 1-like 3 | | NAP1L3 | | 0.0457 |
| 178 | | zinc finger protein 653 | | ZNF653 | | 0.0458 |
| 179 | | mitochondrial E3 ubiquitin protein ligase 1 | | MUL1 | | 0.0458 |
| 180 | | ring finger protein 123 | | RNF123 | | 0.0460 |
| 181 | | HECT and RLD domain containing E3 ubiquitin protein ligase 2 | | HERC2 | | 0.0461 |
| 182 | | POU class 2 homeobox 1 | | POU2F1 | | 0.0464 |
| 183 | | TRAF-interacting protein with forkhead-associated domain | | TIFA | | 0.0465 |
| 184 | | RNA component of mitochondrial RNA processing endoribonuclease | | RMRP | | 0.0466 |
| 185 | | ring finger protein 126 | | RNF126 | | 0.0467 |
| 186 | | dermatan sulfate epimerase-like | | DSEL | | 0.0468 |
| 187 | | heat shock 70kDa protein 1B | | HSPA1B | | 0.0468 |
| 188 | | serpin peptidase inhibitor, clade B (ovalbumin), member 8 | | SERPINB8 | | 0.0471 |
| 189 | | ankyrin repeat and SOCS box containing 14 | | ASB14 | | 0.0473 |
| 190 | | tudor domain containing 12 | | TDRD12 | | 0.0474 |
| 191 | | zinc finger CCHC-type and RNA binding motif 1 | | ZCRB1 | | 0.0476 |
| 192 | | microtubule associated serine/threonine kinase family member 4 | | MAST4 | | 0.0479 |
| 193 | | ZNF667 antisense RNA 1 (head to head) | | ZNF667-AS1 | | 0.0479 |
| 194 | | suppression of tumorigenicity 14 (colon carcinoma) | | ST14 | | 0.0483 |
| 195 | | Yip1 domain family, member 3 | | YIPF3 | | 0.0485 |
| 196 | | SH3 domain binding glutamate-rich protein like 3 | | SH3BGRL3 | | 0.0488 |
| 197 | | chromosome 11 open reading frame 24 | | C11orf24 | | 0.0489 |
| 198 | | coenzyme Q6 monooxygenase | | COQ6 | | 0.0490 |
| 199 | | aminoacyl tRNA synthetase complex-interacting multifunctional | | AIMP2 | | 0.0492 |
| 200 | | RNASEK-C17orf49 readthrough | | RNASEK-C17orf49 | | 0.0497 |
| 201 | | X-ray repair complementing defective repair in Chinese hamster | | XRCC1 | | 0.0499 |

**Supplementary Table 4. List of genes significantly correlated with cortisol blood levels (p-value<0.05)**

|  | **Gene assignment** | | **Gene Symbol** | | **p-value** | |
| --- | --- | --- | --- | --- | --- | --- |
| 1 | | CD69 molecule | | CD69 | | 0.0005 |
| 2 | | period circadian clock 1 | | PER1 | | 0.0005 |
| 3 | | amphiregulin | | AREG | | 0.0006 |
| 4 | | EF-hand calcium binding domain 12 | | EFCAB12 | | 0.0006 |
| 5 | | prenyl (decaprenyl) diphosphate synthase, subunit 1 | | PDSS1 | | 0.0022 |
| 6 | | Spi-C transcription factor (Spi-1/PU.1 related) | | SPIC | | 0.0026 |
| 7 | | advillin | | AVIL | | 0.0028 |
| 8 | | zinc finger CCCH-type containing 12A | | ZC3H12A | | 0.0031 |
| 9 | | oncostatin M | | OSM | | 0.0031 |
| 10 | | transmembrane protein 5 | | TMEM5 | | 0.0033 |
| 11 | | UDP-GlcNAc:betaGal beta-1,3-N-acetylglucosaminyltransfera | | B3GNTL1 | | 0.0034 |
| 12 | | unkempt family zinc finger-like | | UNKL | | 0.0037 |
| 13 | | aldo-keto reductase family 1, member C3 | | AKR1C3 | | 0.0040 |
| 14 | | nudix (nucleoside diphosphate linked moiety X)-type motif 9 | | NUDT9 | | 0.0043 |
| 15 | | DNA-damage-inducible transcript 4 | | DDIT4 | | 0.0044 |
| 16 | | dual specificity phosphatase 1 | | DUSP1 | | 0.0047 |
| 17 | | frequently rearranged in advanced T-cell lymphomas 2 | | FRAT2 | | 0.0050 |
| 18 | | argininosuccinate lyase | | ASL | | 0.0051 |
| 19 | | nuclear factor of kappa light polypeptide gene enhancer in B-cel | | NFKBIA | | 0.0051 |
| 20 | | TSC22 domain family, member 3 | | TSC22D3 | | 0.0052 |
| 21 | | C-type lectin domain family 12, member A | | CLEC12A | | 0.0053 |
| 22 | | histone cluster 1, H2ak | | HIST1H2AK | | 0.0055 |
| 23 | | leucine rich repeat and fibronectin type III domain containing 1 | | LRFN1 | | 0.0060 |
| 24 | | pyrimidinergic receptor P2Y, G-protein coupled, 6 | | P2RY6 | | 0.0060 |
| 25 | | Kruppel-like factor 9 | | KLF9 | | 0.0061 |
| 26 | | histone cluster 1, H1t | | HIST1H1T | | 0.0062 |
| 27 | | TSPY-like 5 | | TSPYL5 | | 0.0064 |
| 28 | | sestrin 1 | | SESN1 | | 0.0066 |
| 29 | | late endosomal/lysosomal adaptor, MAPK and MTOR activator 4 | | LAMTOR4 | | 0.0068 |
| 30 | | glutamine rich 2 | | QRICH2 | | 0.0073 |
| 31 | | Xg blood group | | XG | | 0.0073 |
| 32 | | mannosidase, endo-alpha-like | | MANEAL | | 0.0076 |
| 33 | | chemokine (C-X-C motif) receptor 4 | | CXCR4 | | 0.0095 |
| 34 | | CD19 molecule | | CD19 | | 0.0095 |
| 35 | | ubiquitin protein ligase E3D | | UBE3D | | 0.0096 |
| 36 | | calcitonin-related polypeptide beta | | CALCB | | 0.0102 |
| 37 | | Rab interacting lysosomal protein-like 1 | | RILPL1 | | 0.0102 |
| 38 | | chromosome 12 open reading frame 55 | | C12orf55 | | 0.0105 |
| 39 | | hepatitis A virus cellular receptor 2 | | HAVCR2 | | 0.0105 |
| 40 | | shroom family member 2 | | SHROOM2 | | 0.0106 |
| 41 | | keratin 37 | | KRT37 | | 0.0110 |
| 42 | | kazrin, periplakin interacting protein | | KAZN | | 0.0111 |
| 43 | | interleukin 7 receptor | | IL7R | | 0.0122 |
| 44 | | C-type lectin domain family 6, member A | | CLEC6A | | 0.0123 |
| 45 | | calcium/calmodulin-dependent protein kinase I | | CAMK1 | | 0.0124 |
| 46 | | dynein, axonemal, heavy chain 7 | | DNAH7 | | 0.0124 |
| 47 | | ST8 alpha-N-acetyl-neuraminide alpha-2,8-sialyltransferase 1 | | ST8SIA1 | | 0.0126 |
| 48 | | kinesin family member 3C | | KIF3C | | 0.0127 |
| 49 | | taste receptor, type 2, member 50 | | TAS2R50 | | 0.0128 |
| 50 | | MYC binding protein | | MYCBP | | 0.0130 |
| 51 | | Versican | | VCAN | | 0.0133 |
| 52 | | castor zinc finger 1 | | CASZ1 | | 0.0135 |
| 53 | | MRVI1 antisense RNA 1 | | MRVI1-AS1 | | 0.0135 |
| 54 | | thrombospondin 1 | | THBS1 | | 0.0137 |
| 55 | | WD repeat domain 86 | | WDR86 | | 0.0141 |
| 56 | | RRS1 ribosome biogenesis regulator homolog (S. cerevisiae) | | RRS1 | | 0.0142 |
| 57 | | eukaryotic translation initiation factor 4B | | EIF4B | | 0.0143 |
| 58 | | docking protein 4 | | DOK4 | | 0.0144 |
| 59 | | WD repeat domain 66 | | WDR66 | | 0.0148 |
| 60 | | ZFP69 zinc finger protein B | | ZFP69B | | 0.0150 |
| 61 | | tumor suppressing subtransferable candidate 4 | | TSSC4 | | 0.0151 |
| 62 | | phosphodiesterase 4D interacting protein | | PDE4DIP | | 0.0152 |
| 63 | | sirtuin 6 | | SIRT6 | | 0.0154 |
| 64 | | CACTIN antisense RNA 1 | | CACTIN-AS1 | | 0.0154 |
| 65 | | phosphatidylinositol glycan anchor biosynthesis, class W | | PIGW | | 0.0157 |
| 66 | | V-set and transmembrane domain containing 1 | | VSTM1 | | 0.0158 |
| 67 | | NDRG family member 2 | | NDRG2 | | 0.0158 |
| 68 | | Fibromodulin | | FMOD | | 0.0159 |
| 69 | | notchless homolog 1 (Drosophila) | | NLE1 | | 0.0161 |
| 70 | | tryptophanyl tRNA synthetase 2, mitochondrial | | WARS2 | | 0.0161 |
| 71 | | primary cilia formation | | PIFO | | 0.0162 |
| 72 | | RWD domain containing 2A | | RWDD2A | | 0.0163 |
| 73 | | indoleamine 2,3-dioxygenase 1 | | IDO1 | | 0.0167 |
| 74 | | inner centromere protein antigens 135/155kDa | | INCENP | | 0.0168 |
| 75 | | FK506 binding protein 2, 13kDa | | FKBP2 | | 0.0170 |
| 76 | | syndecan 2 | | SDC2 | | 0.0172 |
| 77 | | solute carrier family 22 (organic cation transporter), member 1 | | SLC22A1 | | 0.0173 |
| 78 | | family with sequence similarity 153, member B | | FAM153B | | 0.0176 |
| 79 | | RNA binding motif protein, X-linked-like 3 | | RBMXL3 | | 0.0180 |
| 80 | | sonic hedgehog | | SHH | | 0.0183 |
| 81 | | chromatin licensing and DNA replication factor 1 | | CDT1 | | 0.0184 |
| 82 | | nuclear transport factor 2-like export factor 1 | | NXT1 | | 0.0185 |
| 83 | | period circadian clock 3 | | PER3 | | 0.0185 |
| 84 | | intersectin 1 (SH3 domain protein) | | ITSN1 | | 0.0186 |
| 85 | | thrombospondin-type laminin G domain and EAR repeats | | TSPEAR | | 0.0186 |
| 86 | | NADH dehydrogenase, subunit 5 (complex I) | | ND5 | | 0.0188 |
| 87 | | discoidin, CUB and LCCL domain containing 1 | | DCBLD1 | | 0.0188 |
| 88 | | cyclin-dependent kinase inhibitor 2A | | CDKN2A | | 0.0189 |
| 89 | | adenosine A3 receptor | | ADORA3 | | 0.0192 |
| 90 | | Meckel syndrome, type 1 | | MKS1 | | 0.0195 |
| 91 | | chordin-like 1 | | CHRDL1 | | 0.0195 |
| 92 | | WD repeat domain 25 | | WDR25 | | 0.0195 |
| 93 | | Fc receptor-like 5 | | FCRL5 | | 0.0201 |
| 94 | | defensin, beta 112 | | DEFB112 | | 0.0202 |
| 95 | | XK, Kell blood group complex subunit-related, X-linked | | XKRX | | 0.0205 |
| 96 | | olfactory receptor, family 8, subfamily G, member 2 | | OR8G2 | | 0.0207 |
| 97 | | FERM, RhoGEF and pleckstrin domain protein 2 | | FARP2 | | 0.0213 |
| 98 | | ferric-chelate reductase 1 | | FRRS1 | | 0.0217 |
| 99 | | zinc finger protein, Y-linked | | ZFY | | 0.0217 |
| 100 | | keratin associated protein 19-6 | | KRTAP19-6 | | 0.0219 |
| 101 | | nuclear cap binding protein subunit 2-like | | NCBP2L | | 0.0222 |
| 102 | | chromosome 6 open reading frame 203 | | C6orf203 | | 0.0224 |
| 103 | | nudix (nucleoside diphosphate linked moiety X)-type motif 4 | | NUDT4 | | 0.0225 |
| 104 | | family with sequence similarity 86, member A | | FAM86A | | 0.0226 |
| 105 | | transmembrane protein 86B | | TMEM86B | | 0.0226 |
| 106 | | growth factor receptor-bound protein 10 | | GRB10 | | 0.0227 |
| 107 | | zinc finger and BTB domain containing 16 | | ZBTB16 | | 0.0229 |
| 108 | | chromosome 5 open reading frame 42 | | C5orf42 | | 0.0230 |
| 109 | | Sad1 and UNC84 domain containing 1 | | SUN1 | | 0.0230 |
| 110 | | myosin light chain kinase 2 | | MYLK2 | | 0.0231 |
| 111 | | doublesex and mab-3 related transcription factor 2 | | DMRT2 | | 0.0231 |
| 112 | | F-box protein 32 | | FBXO32 | | 0.0233 |
| 113 | | serine palmitoyltransferase, long chain base subunit 3 | | SPTLC3 | | 0.0233 |
| 114 | | platelet factor 4 variant 1 | | PF4V1 | | 0.0233 |
| 115 | | UPF3 regulator of nonsense transcripts homolog A (yeast) | | UPF3A | | 0.0235 |
| 116 | | spastic paraplegia 7 (pure and complicated autosomal recessive) | | SPG7 | | 0.0238 |
| 117 | | TAF8 RNA polymerase II, TATA box binding protein (TBP)-associated | | TAF8 | | 0.0238 |
| 118 | | chondroitin sulfate synthase 1 | | CHSY1 | | 0.0242 |
| 119 | | zinc finger protein 610 | | ZNF610 | | 0.0248 |
| 120 | | nudix (nucleoside diphosphate linked moiety X)-type motif 13 | | NUDT13 | | 0.0249 |
| 121 | | dipeptidase 3 | | DPEP3 | | 0.0249 |
| 122 | | 5-hydroxytryptamine (serotonin) receptor 3B, ionotropic | | HTR3B | | 0.0249 |
| 123 | | cholinergic receptor, nicotinic, alpha 10 (neuronal) | | CHRNA10 | | 0.0250 |
| 124 | | wingless-type MMTV integration site family, member 7B | | WNT7B | | 0.0251 |
| 125 | | WAP, follistatin/kazal, immunoglobulin, kunitz and netrin | | WFIKKN2 | | 0.0252 |
| 126 | | phosphatidylinositol glycan anchor biosynthesis, class G | | PIGG | | 0.0257 |
| 127 | | sosondowah ankyrin repeat domain family member D | | SOWAHD | | 0.0258 |
| 128 | | attractin | | ATRN | | 0.0258 |
| 129 | | zinc finger protein 778 | | ZNF778 | | 0.0259 |
| 130 | | cadherin, EGF LAG seven-pass G-type receptor 1 | | CELSR1 | | 0.0259 |
| 131 | | IQ motif containing F2 | | IQCF2 | | 0.0260 |
| 132 | | sortilin 1 | | SORT1 | | 0.0265 |
| 133 | | ferredoxin 1-like | | FDX1L | | 0.0266 |
| 134 | | tripartite motif containing 69 | | TRIM69 | | 0.0266 |
| 135 | | solute carrier family 20 (phosphate transporter), member 2 | | SLC20A2 | | 0.0270 |
| 136 | | exonuclease 5 | | EXO5 | | 0.0271 |
| 137 | | mitochondrially encoded tRNA tyrosine | | MT-TY | | 0.0273 |
| 138 | | sterol carrier protein 2 | | SCP2 | | 0.0275 |
| 139 | | regulator of G-protein signalling 21 | | RGS21 | | 0.0276 |
| 140 | | sperm associated antigen 1 | | SPAG1 | | 0.0279 |
| 141 | | polymerase (RNA) II (DNA directed) polypeptide F | | POLR2F | | 0.0280 |
| 142 | | leucine rich repeat (in FLII) interacting protein 1 | | LRRFIP1 | | 0.0281 |
| 143 | | C-type lectin domain family 12, member B | | CLEC12B | | 0.0282 |
| 144 | | ubiquinol-cytochrome c reductase, Rieske iron-sulfur polypeptid | | UQCRFS1 | | 0.0282 |
| 145 | | leukocyte-associated immunoglobulin-like receptor 1 | | LAIR1 | | 0.0284 |
| 146 | | achaete-scute family bHLH transcription factor 5 | | ASCL5 | | 0.0285 |
| 147 | | integrin, beta 8 | | ITGB8 | | 0.0285 |
| 148 | | tubulin, beta 8 class VIII | | TUBB8 | | 0.0285 |
| 149 | | C-type lectin domain family 4, member E | | CLEC4E | | 0.0286 |
| 150 | | FERM and PDZ domain containing 3 | | FRMPD3 | | 0.0287 |
| 151 | | syntaxin 8 | | STX8 | | 0.0287 |
| 152 | | Sarcospan | | SSPN | | 0.0287 |
| 153 | | ankyrin repeat domain 39 | | ANKRD39 | | 0.0288 |
| 154 | | glutamate dehydrogenase 2 | | GLUD2 | | 0.0292 |
| 155 | | MAX dimerization protein 3 | | MXD3 | | 0.0292 |
| 156 | | GTPase, IMAP family member 2 | | GIMAP2 | | 0.0293 |
| 157 | | activating signal cointegrator 1 complex subunit 1 | | ASCC1 | | 0.0293 |
| 158 | | dehydrogenase/reductase (SDR family) member 13 | | DHRS13 | | 0.0298 |
| 159 | | 5-nucleotidase, cytosolic IIIB | | NT5C3B | | 0.0299 |
| 160 | | olfactory receptor, family 5, subfamily T, member 2 | | OR5T2 | | 0.0300 |
| 161 | | SRY (sex determining region Y)-box 14 | | SOX14 | | 0.0304 |
| 162 | | B-cell translocation gene 1, anti-proliferative | | BTG1 | | 0.0304 |
| 163 | | potassium voltage-gated channel, Shal-related subfamily, member 1 | | KCND1 | | 0.0307 |
| 164 | | POTE ankyrin domain family, member E | | POTEE | | 0.0311 |
| 165 | | tetratricopeptide repeat domain 18 | | TTC18 | | 0.0311 |
| 166 | | Fanconi anemia, complementation group C | | FANCC | | 0.0313 |
| 167 | | adenylate kinase 5 | | AK5 | | 0.0314 |
| 168 | | lectin, galactoside-binding, soluble, 3 | | LGALS3 | | 0.0315 |
| 169 | | zinc finger, CCCH-type with G patch domain | | ZGPAT | | 0.0317 |
| 170 | | 2-phosphoxylose phosphatase 1 | | PXYLP1 | | 0.0317 |
| 171 | | ubiquitin specific peptidase 9, Y-linked | | USP9Y | | 0.0318 |
| 172 | | WW domain binding protein 1 | | WBP1 | | 0.0322 |
| 173 | | eukaryotic translation initiation factor 4A1 | | EIF4A1 | | 0.0322 |
| 174 | | prickle homolog 1 (Drosophila) | | PRICKLE1 | | 0.0322 |
| 175 | | protein tyrosine phosphatase, receptor type, T | | PTPRT | | 0.0326 |
| 176 | | sestrin 2 | | SESN2 | | 0.0326 |
| 177 | | solute carrier family 10, member 5 | | SLC10A5 | | 0.0326 |
| 178 | | THAP domain containing 8 | | THAP8 | | 0.0327 |
| 179 | | sterile alpha and TIR motif containing 1 | | SARM1 | | 0.0328 |
| 180 | | ribosomal protein S4, Y-linked 1 | | RPS4Y1 | | 0.0329 |
| 181 | | calcium regulated heat stable protein 1, 24kDa | | CARHSP1 | | 0.0330 |
| 182 | | leucine rich repeat containing 63 | | LRRC63 | | 0.0332 |
| 183 | | protein serine kinase H2 | | PSKH2 | | 0.0332 |
| 184 | | squalene epoxidase | | SQLE | | 0.0336 |
| 185 | | ligand of numb-protein X 2 | | LNX2 | | 0.0337 |
| 186 | | CD101 molecule | | CD101 | | 0.0337 |
| 187 | | ADAM metallopeptidase domain 23 | | ADAM23 | | 0.0338 |
| 188 | | transforming growth factor, beta 2 | | TGFB2 | | 0.0338 |
| 189 | | A kinase (PRKA) anchor protein 1 | | AKAP1 | | 0.0341 |
| 190 | | microtubule-associated protein 1 light chain 3 alpha | | MAP1LC3A | | 0.0342 |
| 191 | | phosphoinositide-3-kinase, regulatory subunit 3 (gamma) | | PIK3R3 | | 0.0346 |
| 192 | | Recoverin | | RCVRN | | 0.0347 |
| 193 | | spermatogenesis associated 2 | | SPATA2 | | 0.0348 |
| 194 | | collagen, type VIII, alpha 1 | | COL8A1 | | 0.0351 |
| 195 | | chromosome 1 open reading frame 68 | | C1orf68 | | 0.0353 |
| 196 | | IBA57, iron-sulfur cluster assembly homolog (S. cerevisiae) | | IBA57 | | 0.0353 |
| 197 | | apoptosis, caspase activation inhibitor | | AVEN | | 0.0355 |
| 198 | | NAG18 mRNA | | NAG18 | | 0.0357 |
| 199 | | heat shock transcription factor 2 | | HSF2 | | 0.0359 |
| 200 | | ring finger protein 144A | | RNF144A | | 0.0360 |
| 201 | | Obg-like ATPase 1 | | OLA1 | | 0.0360 |
| 202 | | zinc finger and BTB domain containing 17 | | ZBTB17 | | 0.0362 |
| 203 | | heat shock 70kDa protein 6 (HSP70B) | | HSPA6 | | 0.0363 |
| 204 | | adenosine deaminase-like | | ADAL | | 0.0365 |
| 205 | | neuropeptide Y receptor Y5 | | NPY5R | | 0.0366 |
| 206 | | FK506 binding protein 5 | | FKBP5 | | 0.0367 |
| 207 | | abhydrolase domain containing 8 | | ABHD8 | | 0.0369 |
| 208 | | ribonuclease, RNase A family, 9 (non-active) | | RNASE9 | | 0.0370 |
| 209 | | potassium channel tetramerization domain containing 6 | | KCTD6 | | 0.0371 |
| 210 | | aldo-keto reductase family 7-like | | AKR7L | | 0.0371 |
| 211 | | thioesterase superfamily member 4 | | THEM4 | | 0.0376 |
| 212 | | synapse defective 1, Rho GTPase, homolog 2 (C. elegans) | | SYDE2 | | 0.0379 |
| 213 | | tRNA-yW synthesizing protein 5 | | TYW5 | | 0.0381 |
| 214 | | polymerase (RNA) I polypeptide A, 194kDa | | POLR1A | | 0.0382 |
| 215 | | solute carrier family 38, member 7 | | SLC38A7 | | 0.0384 |
| 216 | | family with sequence similarity 174, member A | | FAM174A | | 0.0385 |
| 217 | | v-ets avian erythroblastosis virus E26 oncogene homolog 1 | | ETS1 | | 0.0385 |
| 218 | | solute carrier family 6 (neurotransmitter transporter) | | SLC6A10PB | | 0.0386 |
| 219 | | ceramide synthase 4 | | CERS4 | | 0.0386 |
| 220 | | zinc finger protein 468 | | ZNF468 | | 0.0386 |
| 221 | | cadherin 2, type 1, N-cadherin (neuronal) | | CDH2 | | 0.0387 |
| 222 | | indian hedgehog | | IHH | | 0.0387 |
| 223 | | zinc finger protein 35 | | ZNF35 | | 0.0388 |
| 224 | | G protein-coupled receptor 63 | | GPR63 | | 0.0388 |
| 225 | | family with sequence similarity 153, member B | | FAM153B | | 0.0388 |
| 226 | | membrane-spanning 4-domains, subfamily A, member 13 | | MS4A13 | | 0.0390 |
| 227 | | contactin associated protein-like 5 | | CNTNAP5 | | 0.0390 |
| 228 | | wntless Wnt ligand secretion mediator | | WLS | | 0.0391 |
| 229 | | hydroxysteroid (11-beta) dehydrogenase 2 | | HSD11B2 | | 0.0392 |
| 230 | | tumor necrosis factor, alpha-induced protein 3 | | TNFAIP3 | | 0.0394 |
| 231 | | zinc finger protein 362 | | ZNF362 | | 0.0395 |
| 232 | | dual specificity phosphatase 28 | | DUSP28 | | 0.0397 |
| 233 | | 2-oxoglutarate and iron-dependent oxygenase domain containing 1 | | OGFOD1 | | 0.0397 |
| 234 | | polypeptide N-acetylgalactosaminyltransferase 14 | | GALNT14 | | 0.0402 |
| 235 | | orthodenticle homeobox 1 | | OTX1 | | 0.0403 |
| 236 | | ubiquitin-conjugating enzyme E2D 2 | | UBE2D2 | | 0.0403 |
| 237 | | alkaline ceramidase 3 | | ACER3 | | 0.0404 |
| 238 | | potassium voltage-gated channel, subfamily H (eag-related) | | KCNH5 | | 0.0406 |
| 239 | | chromosome 14 open reading frame 142 | | C14orf142 | | 0.0408 |
| 240 | | zinc finger protein 333 | | ZNF333 | | 0.0409 |
| 241 | | guanine nucleotide binding protein (G protein), gamma 7 | | GNG7 | | 0.0409 |
| 242 | | ISL LIM homeobox 1 | | ISL1 | | 0.0412 |
| 243 | | amino-terminal enhancer of split | | AES | | 0.0412 |
| 244 | | solute carrier family 6 (neurotransmitter transporter), member 4 | | SLC6A4 | | 0.0413 |
| 245 | | TXK tyrosine kinase | | TXK | | 0.0414 |
| 246 | | solute carrier family 35 (UDP-galactose transporter) | | SLC35A2 | | 0.0417 |
| 247 | | keratin associated protein 27-1 | | KRTAP27-1 | | 0.0419 |
| 248 | | atonal homolog 7 (Drosophila) | | ATOH7 | | 0.0422 |
| 249 | | cleavage and polyadenylation factor I subunit 1 | | CLP1 | | 0.0423 |
| 250 | | tyrosine 3-monooxygenase/tryptophan 5-monooxygenase activation | | YWHAH | | 0.0424 |
| 251 | | TNFRSF1A-associated via death domain | | TRADD | | 0.0426 |
| 252 | | HEAT repeat containing 3 | | HEATR3 | | 0.0427 |
| 253 | | chromosome 1 open reading frame 74 | | C1orf74 | | 0.0432 |
| 254 | | keratin associated protein 13-3 | | KRTAP13-3 | | 0.0433 |
| 255 | | t-complex 10-like | | TCP10L | | 0.0433 |
| 256 | | OTU deubiquitinase 7B | | OTUD7B | | 0.0435 |
| 257 | | zinc finger protein 879 | | ZNF879 | | 0.0438 |
| 258 | | MCF.2 cell line derived transforming sequence | | MCF2 | | 0.0439 |
| 259 | | thioredoxin interacting protein | | TXNIP | | 0.0440 |
| 260 | | BMX non-receptor tyrosine kinase | | BMX | | 0.0442 |
| 261 | | BEN domain containing 4 | | BEND4 | | 0.0444 |
| 262 | | chemokine (C-X-C motif) ligand 1 | | CXCL1 | | 0.0444 |
| 263 | | eukaryotic translation initiation factor 5A-like 1 | | EIF5AL1 | | 0.0446 |
| 264 | | tumor necrosis factor (ligand) superfamily, member 8 | | TNFSF8 | | 0.0447 |
| 265 | | SPC24, NDC80 kinetochore complex component | | SPC24 | | 0.0448 |
| 266 | | insulin-like growth factor binding protein 3 | | IGFBP3 | | 0.0448 |
| 267 | | ubiquitously transcribed tetratricopeptide | | UTY | | 0.0449 |
| 268 | | glycosyltransferase-like 1B | | GYLTL1B | | 0.0449 |
| 269 | | enolase 1, (alpha) | | ENO1 | | 0.0450 |
| 270 | | transmembrane protein 99 | | TMEM99 | | 0.0451 |
| 271 | | chondrosarcoma associated gene 1 | | CSAG1 | | 0.0454 |
| 272 | | gamma-secretase activating protein | | GSAP | | 0.0455 |
| 273 | | DiGeorge syndrome critical region gene 6-like | | DGCR6L | | 0.0457 |
| 274 | | zinc ribbon domain containing 1 | | ZNRD1 | | 0.0458 |
| 275 | | myomesin 1 | | MYOM1 | | 0.0458 |
| 276 | | v-myc avian myelocytomatosis viral oncogene homolog | | MYC | | 0.0459 |
| 277 | | Somatostatin | | SST | | 0.0460 |
| 278 | | kelch repeat and BTB (POZ) domain containing 6 | | KBTBD6 | | 0.0460 |
| 279 | | heat shock protein, alpha-crystallin-related, B9 | | HSPB9 | | 0.0461 |
| 280 | | AF4/FMR2 family, member 2 | | AFF2 | | 0.0461 |
| 281 | | BRICK1, SCAR/WAVE actin-nucleating complex subunit | | BRK1 | | 0.0461 |
| 282 | | lysine (K)-specific demethylase 4D | | KDM4D | | 0.0461 |
| 283 | | family with sequence similarity 47, member A | | FAM47A | | 0.0462 |
| 284 | | protein phosphatase 1, regulatory (inhibitor) subunit 14A | | PPP1R14A | | 0.0464 |
| 285 | | guanine nucleotide binding protein (G protein) | | GNAI1 | | 0.0464 |
| 286 | | zona pellucida binding protein | | ZPBP | | 0.0465 |
| 287 | | SRY (sex determining region Y)-box 18 | | SOX18 | | 0.0465 |
| 288 | | integrin, beta 4 | | ITGB4 | | 0.0466 |
| 289 | | polymerase (RNA) III (DNA directed) polypeptide G (32kD)-like | | POLR3GL | | 0.0466 |
| 290 | | deafness, autosomal dominant 5 | | DFNA5 | | 0.0469 |
| 291 | | ketohexokinase (fructokinase) | | KHK | | 0.0471 |
| 292 | | COP9 signalosome subunit 8 | | COPS8 | | 0.0472 |
| 293 | | Myoferlin | | MYOF | | 0.0474 |
| 294 | | sterol O-acyltransferase 2 | | SOAT2 | | 0.0474 |
| 295 | | zinc finger protein 571 | | ZNF571 | | 0.0476 |
| 296 | | neuromedin B | | NMB | | 0.0478 |
| 297 | | G protein-coupled receptor 68 | | GPR68 | | 0.0478 |
| 298 | | SHC SH2-domain binding protein 1-like | | SHCBP1L | | 0.0478 |
| 299 | | GATA binding protein 2 | | GATA2 | | 0.0482 |
| 300 | | dermatan sulfate epimerase-like | | DSEL | | 0.0483 |
| 301 | | chromosome 1 open reading frame 100 | | C1orf100 | | 0.0483 |
| 302 | | WW domain containing oxidoreductase | | WWOX | | 0.0484 |
| 303 | | fibrillarin-like 1 | | FBLL1 | | 0.0484 |
| 304 | | CASK interacting protein 1 | | CASKIN1 | | 0.0485 |
| 305 | | C1RL antisense RNA 1 | | C1RL-AS1 | | 0.0486 |
| 306 | | P antigen family, member 4 (prostate associated) | | PAGE4 | | 0.0487 |
| 307 | | matrix metallopeptidase 24 (membrane-inserted) | | MMP24 | | 0.0487 |
| 308 | | ring finger protein 144B | | RNF144B | | 0.0488 |
| 309 | | carcinoembryonic antigen-related cell adhesion molecule 19 | | CEACAM19 | | 0.0490 |
| 310 | | T cell receptor gamma variable 3 | | TRGV3 | | 0.0491 |
| 311 | | Cbp/p300-interacting transactivator, with Glu/Asp-rich carboxy-t | | CITED4 | | 0.0491 |
| 312 | | eukaryotic translation initiation factor 2 | | EIF2S2P4 | | 0.0493 |
| 313 | | family with sequence similarity 92, member A3 | | FAM92A1P2 | | 0.0494 |
| 314 | | inscuteable homolog (Drosophila) | | INSC | | 0.0495 |
| 315 | | neural cell adhesion molecule 1 | | NCAM1 | | 0.0495 |
| 316 | | peroxisomal biogenesis factor 6 | | PEX6 | | 0.0496 |
| 317 | | zinc finger protein 597 | | ZNF597 | | 0.0496 |
| 318 | | pleckstrin homology-like domain, family A, member 1 | | PHLDA1 | | 0.0497 |
| 319 | | testis expressed 101 | | TEX101 | | 0.0498 |
| 320 | | lysine (K)-specific demethylase 5D | | KDM5D | | 0.0499 |
| 321 | | tumor protein p63 regulated 1 | | TPRG1 | | 0.0499 |

**Supplementary Table 5. List of genes significantly correlated with telomere length reduction (p- value<0.05)**

|  | **Gene assignment** | **Gene Symbol** | **p-value** |
| --- | --- | --- | --- |
| 1 | G protein-coupled receptor 125 | GPR125 | 0.0007 |
| 2 | t-complex 1 | TCP1 | 0.0007 |
| 3 | T-box 19 | TBX19 | 0.0010 |
| 4 | 2-oxoglutarate and iron-dependent oxygenase domain containing 2 | OGFOD2 | 0.0014 |
| 5 | CASP2 and RIPK1 domain containing adaptor with death domain | CRADD | 0.0015 |
| 6 | connector enhancer of kinase suppressor of Ras 2 | CNKSR2 | 0.0017 |
| 7 | taxilin gamma | TXLNG | 0.0018 |
| 8 | TPX2, microtubule-associated, homolog (Xenopus laevis) | TPX2 | 0.0020 |
| 9 | nuclear distribution C homolog (A. nidulans) | NUDC | 0.0022 |
| 10 | inositol polyphosphate-5-phosphatase | INPP5B | 0.0023 |
| 11 | fructosamine 3 kinase related protein | FN3KRP | 0.0026 |
| 12 | aquaporin 1 (Colton blood group) | AQP1 | 0.0028 |
| 13 | chromosome 1 open reading frame 85 | C1orf85 | 0.0030 |
| 14 | charged multivesicular body protein 7 | CHMP7 | 0.0031 |
| 15 | GRB2-binding adaptor protein, transmembrane | GAPT | 0.0034 |
| 16 | corticotropin releasing hormone binding protein | CRHBP | 0.0035 |
| 17 | SH3KBP1 binding protein 1 | SHKBP1 | 0.0036 |
| 18 | COMM domain containing 5 | COMMD5 | 0.0037 |
| 19 | block of proliferation 1 | BOP1 | 0.0038 |
| 20 | synaptogyrin 2 | SYNGR2 | 0.0038 |
| 21 | keratin associated protein 20-1 | KRTAP20-1 | 0.0042 |
| 22 | aldehyde dehydrogenase 3 family, member A2 | ALDH3A2 | 0.0045 |
| 23 | minichromosome maintenance complex component 4 | MCM4 | 0.0047 |
| 24 | sodium channel, voltage-gated, type VII, alpha subunit | SCN7A | 0.0047 |
| 25 | excision repair cross-complementing rodent repair deficiency | ERCC3 | 0.0050 |
| 26 | taste receptor, type 2, member 40 | TAS2R40 | 0.0052 |
| 27 | ATP/GTP binding protein-like 5 | AGBL5 | 0.0053 |
| 28 | potassium voltage-gated channel, Isk-related family, member 1 | KCNE1 | 0.0054 |
| 29 | topoisomerase (DNA) I, mitochondrial | TOP1MT | 0.0056 |
| 30 | neurobeachin | NBEA | 0.0056 |
| 31 | tRNA 5-methylaminomethyl-2-thiouridylate methyltransferase | TRMU | 0.0057 |
| 32 | armadillo repeat containing 2 | ARMC2 | 0.0057 |
| 33 | tropomyosin 3 | TPM3 | 0.0059 |
| 34 | chromosome 10 open reading frame 2 | C10orf2 | 0.0059 |
| 35 | alcohol dehydrogenase 1A (class I), alpha polypeptide | ADH1A | 0.0059 |
| 36 | solute carrier family 11 | SLC11A1 | 0.0061 |
| 37 | phosphoinositide-3-kinase interacting protein 1 | PIK3IP1 | 0.0062 |
| 38 | ankyrin repeat domain 30B | ANKRD30B | 0.0063 |
| 39 | ATP-binding cassette, sub-family C (CFTR/MRP), member 2 | ABCC2 | 0.0066 |
| 40 | U2 small nuclear RNA auxiliary factor 1-like 4 | U2AF1L4 | 0.0068 |
| 41 | olfactory receptor, family 13, subfamily H, member 1 | OR13H1 | 0.0068 |
| 42 | solute carrier family 26, member 8 | SLC26A8 | 0.0069 |
| 43 | heterogeneous nuclear ribonucleoprotein A3 | HNRNPA3 | 0.0069 |
| 44 | C-type lectin domain family 1, member A | CLEC1A | 0.0071 |
| 45 | ribosomal RNA processing 15 homolog (S. cerevisiae) | RRP15 | 0.0072 |
| 46 | translocase of inner mitochondrial membrane 50 homolog | TIMM50 | 0.0073 |
| 47 | dedicator of cytokinesis 10 | DOCK10 | 0.0076 |
| 48 | serine peptidase inhibitor, Kunitz type, 2 | SPINT2 | 0.0076 |
| 49 | GH3 domain containing | GHDC | 0.0079 |
| 50 | eukaryotic translation initiation factor 3, subunit B | EIF3B | 0.0081 |
| 51 | RAB3A interacting protein (rabin3) | RAB3IP | 0.0081 |
| 52 | chromosome 3 open reading frame 37 | C3orf37 | 0.0082 |
| 53 | GNAS complex locus | GNAS | 0.0085 |
| 54 | Rh-associated glycoprotein | RHAG | 0.0087 |
| 55 | ribosomal protein L39-like | RPL39L | 0.0089 |
| 56 | forkhead box P1 | FOXP1 | 0.0097 |
| 57 | interleukin 1, alpha | IL1A | 0.0101 |
| 58 | UDP-GlcNAc:betaGal beta-1,3-N-acetylglucosaminyltransferase 8 | B3GNT8 | 0.0103 |
| 59 | SPG20 opposite strand | SPG20OS | 0.0103 |
| 60 | regulator of G-protein signalling 3 | RGS3 | 0.0105 |
| 61 | serine/arginine-rich splicing factor 4 | SRSF4 | 0.0105 |
| 62 | chromosome 11 open reading frame 63 | C11orf63 | 0.0106 |
| 63 | tRNA-yW synthesizing protein 3 homolog (S. cerevisiae) | TYW3 | 0.0110 |
| 64 | glycine-N-acyltransferase-like 1 | GLYATL1 | 0.0111 |
| 65 | leukocyte-associated immunoglobulin-like receptor 1 | LAIR1 | 0.0112 |
| 66 | leukocyte immunoglobulin-like receptor, subfamily A | LILRA3 | 0.0112 |
| 67 | unkempt homolog (Drosophila)-like | UNKL | 0.0115 |
| 68 | lysophosphatidylcholine acyltransferase 4 | LPCAT4 | 0.0121 |
| 69 | pannexin 1 | PANX1 | 0.0122 |
| 70 | FOS-like antigen 2 | FOSL2 | 0.0127 |
| 71 | adducin 2 (beta) | ADD2 | 0.0129 |
| 72 | amyloid beta (A4) precursor protein-binding, family A, member 2 | APBA2 | 0.0129 |
| 73 | TCF3 (E2A) fusion partner (in childhood Leukemia) | TFPT | 0.0129 |
| 74 | transmembrane protein 205 | TMEM205 | 0.0130 |
| 75 | trinucleotide repeat containing 18 | TNRC18 | 0.0131 |
| 76 | amyloid beta (A4) precursor protein-binding, family B, member 1 | APBB1 | 0.0131 |
| 77 | N(alpha)-acetyltransferase 30, NatC catalytic subunit | NAA30 | 0.0131 |
| 78 | GTPase activating protein (SH3 domain) binding protein 1 | G3BP1 | 0.0133 |
| 79 | lipid phosphate phosphatase-related protein type 5 | LPPR5 | 0.0134 |
| 80 | coiled-coil domain containing 79 | CCDC79 | 0.0134 |
| 81 | ectodysplasin A receptor | EDAR | 0.0135 |
| 82 | TruB pseudouridine (psi) synthase homolog 2 (E. coli) | TRUB2 | 0.0136 |
| 83 | LysM, putative peptidoglycan-binding, domain containing 4 | LYSMD4 | 0.0136 |
| 84 | guanine nucleotide binding protein (G protein) | GNAO1 | 0.0137 |
| 85 | melanoma inhibitory activity 2 | MIA2 | 0.0139 |
| 86 | chromosome 6 open reading frame 108 | C6orf108 | 0.0141 |
| 87 | HCLS1 binding protein 3 | HS1BP3 | 0.0141 |
| 88 | dipeptidase 3 | DPEP3 | 0.0141 |
| 89 | thymosin beta 4, X-linked | TMSB4X | 0.0142 |
| 90 | inturned planar cell polarity effector homolog (Drosophila) | INTU | 0.0144 |
| 91 | chromosome 9 open reading frame 3 | C9orf3 | 0.0145 |
| 92 | HOXA cluster antisense RNA 2 (non-protein coding) | HOXA-AS2 | 0.0145 |
| 93 | LIM domain 7 | LMO7 | 0.0147 |
| 94 | FERM, RhoGEF and pleckstrin domain protein 2 | FARP2 | 0.0147 |
| 95 | thyroid hormone receptor interactor 10 | TRIP10 | 0.0147 |
| 96 | eyes shut homolog (Drosophila) | EYS | 0.0147 |
| 97 | acid-sensing (proton-gated) ion channel 3 | ASIC3 | 0.0148 |
| 98 | spermine synthase | SMS | 0.0148 |
| 99 | runt-related transcription factor 1 | RUNX1 | 0.0148 |
| 100 | BTB and CNC homology 1, basic leucine zipper transcription factor | BACH2 | 0.0148 |
| 101 | solute carrier family 19 (folate transporter), member 1 | SLC19A1 | 0.0150 |
| 102 | NEL-like 2 (chicken) | NELL2 | 0.0154 |
| 103 | hippocampus abundant transcript-like 1 | HIATL1 | 0.0154 |
| 104 | G protein-coupled receptor 97 | GPR97 | 0.0159 |
| 105 | motor neuron and pancreas homeobox 1 | MNX1 | 0.0159 |
| 106 | ATPase, Na+/K+ transporting, alpha 1 polypeptide | ATP1A1 | 0.0162 |
| 107 | crystallin, gamma S | CRYGS | 0.0163 |
| 108 | DnaJ (Hsp40) homolog, subfamily B, member 5 | DNAJB5 | 0.0164 |
| 109 | nuclear factor of kappa light polypeptide gene enhancer in B-cells | NFKB2 | 0.0164 |
| 110 | centrosomal protein 41kDa | CEP41 | 0.0164 |
| 111 | UDP-N-acetyl-alpha-D-galactosamine:polypeptide N-acetylgalactosa | GALNT12 | 0.0167 |
| 112 | fibrous sheath interacting protein 1 | FSIP1 | 0.0168 |
| 113 | synovial sarcoma, X breakpoint 9 | SSX9 | 0.0169 |
| 114 | heterogeneous nuclear ribonucleoprotein K | HNRNPK | 0.0169 |
| 115 | ring finger protein 5, E3 ubiquitin protein ligase | RNF5 | 0.0170 |
| 116 | small EDRK-rich factor 1A (telomeric) | SERF1A | 0.0171 |
| 117 | chromosome 6 open reading frame 1 | C6orf1 | 0.0171 |
| 118 | hemoglobin, alpha 1 | HBA1 | 0.0172 |
| 119 | high mobility group nucleosomal binding domain 2 | HMGN2 | 0.0172 |
| 120 | short chain dehydrogenase/reductase family 42E, member 1 | SDR42E1 | 0.0175 |
| 121 | UTP14, U3 small nucleolar ribonucleoprotein, homolog A (yeast) | UTP14A | 0.0176 |
| 122 | cappuccino homolog (mouse) | CNO | 0.0176 |
| 123 | activin A receptor, type IC | ACVR1C | 0.0182 |
| 124 | transmembrane protein 66 | TMEM66 | 0.0183 |
| 125 | nuclear factor of kappa light polypeptide gene enhancer in B-cells | NFKBIE | 0.0186 |
| 126 | cyclin G2 | CCNG2 | 0.0187 |
| 127 | spleen tyrosine kinase | SYK | 0.0187 |
| 128 | interleukin-1 receptor-associated kinase 2 | IRAK2 | 0.0188 |
| 129 | basic leucine zipper and W2 domains 2 | BZW2 | 0.0190 |
| 130 | cytokine induced apoptosis inhibitor 1 | CIAPIN1 | 0.0192 |
| 131 | cell cycle associated protein 1 | CAPRIN1 | 0.0192 |
| 132 | transmembrane emp24 protein transport domain containing 3 | TMED3 | 0.0193 |
| 133 | WW domain containing adaptor with coiled-coil | WAC | 0.0193 |
| 134 | CCR4-NOT transcription complex, subunit 10 | CNOT10 | 0.0196 |
| 135 | proprotein convertase subtilisin/kexin type 1 inhibitor | PCSK1N | 0.0197 |
| 136 | coiled-coil domain containing 36 | CCDC36 | 0.0198 |
| 137 | presequence translocase-associated motor 16 homolog | PAM16 | 0.0198 |
| 138 | DEAD (Asp-Glu-Ala-Asp) box helicase 5 | DDX5 | 0.0200 |
| 139 | haptoglobin-related protein | HPR | 0.0202 |
| 140 | exocyst complex component 3 | EXOC3 | 0.0202 |
| 141 | proliferation-associated 2G4, 38kDa | PA2G4 | 0.0202 |
| 142 | Yae1 domain containing 1 | YAE1D1 | 0.0203 |
| 143 | solute carrier family 25 (mitochondrial thiamine pyrophosphate) | SLC25A19 | 0.0203 |
| 144 | synaptotagmin binding, cytoplasmic RNA interacting protein | SYNCRIP | 0.0204 |
| 145 | kinesin family member 3B | KIF3B | 0.0208 |
| 146 | sorting nexin 7 | SNX7 | 0.0208 |
| 147 | proteolipid protein 2 (colonic epithelium-enriched) | PLP2 | 0.0209 |
| 148 | RNA, U4atac small nuclear (U12-dependent splicing) | RNU4ATAC | 0.0209 |
| 149 | CCCTC-binding factor (zinc finger protein) | CTCF | 0.0211 |
| 150 | small glutamine-rich tetratricopeptide repeat (TPR) | SGTB | 0.0211 |
| 151 | acid phosphatase 1, soluble | ACP1 | 0.0211 |
| 152 | butyrophilin-like 8 | BTNL8 | 0.0211 |
| 153 | Rho GTPase activating protein 44 | ARHGAP44 | 0.0212 |
| 154 | Rap guanine nucleotide exchange factor (GEF) 6 | RAPGEF6 | 0.0215 |
| 155 | copper chaperone for superoxide dismutase | CCS | 0.0216 |
| 156 | death-associated protein kinase 2 | DAPK2 | 0.0216 |
| 157 | WD repeat domain 5 | WDR5 | 0.0217 |
| 158 | phosphatidic acid phosphatase type 2A | PPAP2A | 0.0217 |
| 159 | testis-specific serine kinase substrate | TSKS | 0.0220 |
| 160 | small proline-rich protein 2B | SPRR2B | 0.0223 |
| 161 | solute carrier family 22, member 18 | SLC22A18 | 0.0223 |
| 162 | chitinase 3-like 2 | CHI3L2 | 0.0225 |
| 163 | glucose-fructose oxidoreductase domain containing 1 | GFOD1 | 0.0226 |
| 164 | RGM domain family, member B | RGMB | 0.0226 |
| 165 | pregnancy specific beta-1-glycoprotein 2 | PSG2 | 0.0227 |
| 166 | ubiquilin 4 | UBQLN4 | 0.0228 |
| 167 | protein tyrosine phosphatase, mitochondrial 1 | PTPMT1 | 0.0228 |
| 168 | ribosomal protein L13a | RPL13A | 0.0228 |
| 169 | extended synaptotagmin-like protein 2 | ESYT2 | 0.0229 |
| 170 | B and T lymphocyte associated | BTLA | 0.0231 |
| 171 | polyhomeotic homolog 2 (Drosophila) | PHC2 | 0.0232 |
| 172 | tetraspanin 4 | TSPAN4 | 0.0233 |
| 173 | killer cell immunoglobulin-like receptor, three domains | KIR3DL2 | 0.0233 |
| 174 | tropomodulin 4 (muscle) | TMOD4 | 0.0235 |
| 175 | neutrophil cytosolic factor 4 | NCF4 | 0.0237 |
| 176 | TAR DNA binding protein | TARDBP | 0.0238 |
| 177 | coiled-coil-helix-coiled-coil-helix domain containing 10 | CHCHD10 | 0.0241 |
| 178 | solute carrier family 35, member E3 | SLC35E3 | 0.0242 |
| 179 | protein tyrosine phosphatase, non-receptor type 13 | PTPN13 | 0.0243 |
| 180 | prickle homolog 4 (Drosophila) | PRICKLE4 | 0.0245 |
| 181 | RE1-silencing transcription factor | REST | 0.0245 |
| 182 | UFM1-specific peptidase 1 (non-functional) | UFSP1 | 0.0246 |
| 183 | heterogeneous nuclear ribonucleoprotein M | HNRNPM | 0.0246 |
| 184 | general transcription factor IIIC, polypeptide 4 | GTF3C4 | 0.0247 |
| 185 | parvalbumin | PVALB | 0.0250 |
| 186 | v-abl Abelson murine leukemia viral oncogene homolog 2 | ABL2 | 0.0251 |
| 187 | RAB1B, member RAS oncogene family | RAB1B | 0.0254 |
| 188 | ring finger protein, transmembrane 1 | RNFT1 | 0.0255 |
| 189 | succinate dehydrogenase complex, subunit A, flavoprotein (Fp) | SDHA | 0.0258 |
| 190 | solute carrier family 7 | SLC7A6 | 0.0260 |
| 191 | solute carrier family 26, member 11 | SLC26A11 | 0.0260 |
| 192 | actin binding LIM protein 1 | ABLIM1 | 0.0262 |
| 193 | transmembrane protease, serine 11B | TMPRSS11B | 0.0266 |
| 194 | TNF receptor-associated factor 3 interacting protein 1 | TRAF3IP1 | 0.0266 |
| 195 | potassium channel tetramerisation domain containing 9 | KCTD9 | 0.0266 |
| 196 | polymerase (RNA) I polypeptide E | POLR1E | 0.0267 |
| 197 | histone cluster 1, H2bn | HIST1H2BN | 0.0268 |
| 198 | chromosome 3 open reading frame 62 | C3orf62 | 0.0268 |
| 199 | Fanconi anemia, complementation group E | FANCE | 0.0271 |
| 200 | meiosis-specific nuclear structural 1 | MNS1 | 0.0271 |
| 201 | NFS1 nitrogen fixation 1 homolog (S. cerevisiae) | NFS1 | 0.0272 |
| 202 | methylmalonic aciduria (cobalamin deficiency) cblB type | MMAB | 0.0276 |
| 203 | olfactory receptor, family 5, subfamily K, member 1 | OR5K1 | 0.0276 |
| 204 | oxysterol binding protein-like 10 | OSBPL10 | 0.0279 |
| 205 | transmembrane protein 161B | TMEM161B | 0.0281 |
| 206 | glutathione S-transferase pi 1 | GSTP1 | 0.0281 |
| 207 | ribosomal protein L26-like 1 | RPL26L1 | 0.0284 |
| 208 | sex comb on midleg-like 1 (Drosophila) | SCML1 | 0.0284 |
| 209 | dehydrogenase/reductase (SDR family) member 7B | DHRS7B | 0.0284 |
| 210 | myeloid/lymphoid or mixed-lineage leukemia | MLL | 0.0286 |
| 211 | interferon, alpha 8 | IFNA8 | 0.0286 |
| 212 | NOP2/Sun domain family, member 5 | NSUN5 | 0.0286 |
| 213 | Rho guanine nucleotide exchange factor (GEF) 5 | ARHGEF5 | 0.0287 |
| 214 | coiled-coil domain containing 170 | CCDC170 | 0.0287 |
| 215 | vestigial like 3 (Drosophila) | VGLL3 | 0.0289 |
| 216 | small nuclear ribonucleoprotein polypeptide B | SNRPB2 | 0.0289 |
| 217 | killer cell immunoglobulin-like receptor, two domains | KIR2DL4 | 0.0289 |
| 218 | polycystic kidney disease 2-like 2 | PKD2L2 | 0.0290 |
| 219 | alanyl (membrane) aminopeptidase | ANPEP | 0.0290 |
| 220 | chromosome 16 open reading frame 5 | C16orf5 | 0.0291 |
| 221 | phosphatidylinositol-5-phosphate 4-kinase, type II, beta | PIP4K2B | 0.0291 |
| 222 | olfactory receptor, family 6, subfamily C, member 6 | OR6C6 | 0.0292 |
| 223 | spectrin, beta, non-erythrocytic 1 | SPTBN1 | 0.0292 |
| 224 | H1 histone family, member 0 | H1F0 | 0.0294 |
| 225 | ribokinase | RBKS | 0.0295 |
| 226 | KIAA0319 | KIAA0319 | 0.0296 |
| 227 | RNA, U6 small nuclear 71 | RNU6-71 | 0.0296 |
| 228 | speedy homolog E1 (Xenopus laevis) | SPDYE1 | 0.0297 |
| 229 | ubiquitin-conjugating enzyme E2K | UBE2K | 0.0300 |
| 230 | adenosylhomocysteinase-like 1 | AHCYL1 | 0.0303 |
| 231 | ribosomal RNA processing 12 homolog (S. cerevisiae) | RRP12 | 0.0303 |
| 232 | chromosome 22 open reading frame 43 | C22orf43 | 0.0303 |
| 233 | patatin-like phospholipase domain containing 1 | PNPLA1 | 0.0305 |
| 234 | chromosome 16 open reading frame 88 | C16orf88 | 0.0309 |
| 235 | chromosome 9 open reading frame 9 | C9orf9 | 0.0309 |
| 236 | ubiquitin-fold modifier 1 | UFM1 | 0.0310 |
| 237 | NYN domain and retroviral integrase containing | NYNRIN | 0.0310 |
| 238 | IMP4, U3 small nucleolar ribonucleoprotein, homolog (yeast) | IMP4 | 0.0312 |
| 239 | mesoderm specific transcript homolog (mouse) | MEST | 0.0313 |
| 240 | chromosome 11 open reading frame 31 | C11orf31 | 0.0318 |
| 241 | serine/threonine kinase 11 interacting protein | STK11IP | 0.0318 |
| 242 | sperm associated antigen 16 | SPAG16 | 0.0319 |
| 243 | RUN and SH3 domain containing 2 | RUSC2 | 0.0320 |
| 244 | Fc receptor-like 5 | FCRL5 | 0.0322 |
| 245 | CD177 molecule | CD177 | 0.0324 |
| 246 | chromosome 16 open reading frame 80 | C16orf80 | 0.0325 |
| 247 | chromosome 19 open reading frame 48 | C19orf48 | 0.0325 |
| 248 | ATP-binding cassette, sub-family B (MDR/TAP), member 10 | ABCB10 | 0.0328 |
| 249 | chromosome 13 open reading frame 33 | C13orf33 | 0.0328 |
| 250 | DNA cross-link repair 1B | DCLRE1B | 0.0330 |
| 251 | Yip1 interacting factor homolog B (S. cerevisiae) | YIF1B | 0.0330 |
| 252 | lipid phosphate phosphatase-related protein type 2 | LPPR2 | 0.0330 |
| 253 | EF-hand domain family, member A2 | EFHA2 | 0.0330 |
| 254 | pitrilysin metallopeptidase 1 | PITRM1 | 0.0331 |
| 255 | leucine-rich alpha-2-glycoprotein 1 | LRG1 | 0.0332 |
| 256 | transcription factor NF-E4 | NFE4 | 0.0333 |
| 257 | ST6 beta-galactosamide alpha-2,6-sialyltranferase 2 | ST6GAL2 | 0.0334 |
| 258 | ras homolog family member H | RHOH | 0.0335 |
| 259 | FLJ45950 protein | FLJ45950 | 0.0336 |
| 260 | solute carrier family 22 (organic cation/carnitine transporter) | SLC22A16 | 0.0341 |
| 261 | excision repair cross-complementing rodent repair deficiency | ERCC6L | 0.0342 |
| 262 | integrin, alpha 8 | ITGA8 | 0.0344 |
| 263 | dolichyl-phosphate (UDP-N-acetylglucosamine) | DPAGT1 | 0.0345 |
| 264 | ubiquitin specific peptidase 11 | USP11 | 0.0345 |
| 265 | F-box protein 41 | FBXO41 | 0.0345 |
| 266 | B-cell translocation gene 4 | BTG4 | 0.0345 |
| 267 | killer cell immunoglobulin-like receptor | KIR3DL3 | 0.0346 |
| 268 | nuclear casein kinase and cyclin-dependent kinase substrate 1 | NUCKS1 | 0.0346 |
| 269 | ENST00000194097 // NAIP // NLR family, apoptosis inhibitory protein | NAIP | 0.0347 |
| 270 | ì Sfi1 homolog, spindle assembly associated (yeast) | SFI1 | 0.0347 |
| 271 | arginine decarboxylase | ADC | 0.0348 |
| 272 | cytotoxic and regulatory T cell molecule | CRTAM | 0.0349 |
| 273 | prostaglandin F receptor (FP) | PTGFR | 0.0351 |
| 274 | coiled-coil domain containing 166 | CCDC166 | 0.0353 |
| 275 | PERP, TP53 apoptosis effector | PERP | 0.0353 |
| 276 | RAB42, member RAS oncogene family | RAB42 | 0.0355 |
| 277 | protocadherin 12 | PCDH12 | 0.0355 |
| 278 | 1-acylglycerol-3-phosphate O-acyltransferase 1 | AGPAT1 | 0.0355 |
| 279 | myeloid/lymphoid or mixed-lineage leukemia | MLLT3 | 0.0355 |
| 280 | leukocyte immunoglobulin-like receptor, subfamily A | LILRA5 | 0.0355 |
| 281 | tripartite motif containing 28 | TRIM28 | 0.0358 |
| 282 | glycerophosphodiester phosphodiesterase domain containing 3 | GDPD3 | 0.0359 |
| 283 | heterogeneous nuclear ribonucleoprotein D | HNRNPD | 0.0359 |
| 284 | solute carrier family 2 (facilitated glucose transporter) | SLC2A13 | 0.0360 |
| 285 | chromosome 10 open reading frame 125 | C10orf125 | 0.0360 |
| 286 | F-box protein 31 | FBXO31 | 0.0361 |
| 287 | solute carrier family 16, member 4 | SLC16A4 | 0.0361 |
| 288 | glycerol-3-phosphate acyltransferase, mitochondrial | GPAM | 0.0362 |
| 289 | chromosome 12 open reading frame 52 | C12orf52 | 0.0366 |
| 290 | cyclin Y-like 1 | CCNYL1 | 0.0366 |
| 291 | golgin A7 | GOLGA7 | 0.0372 |
| 292 | myocyte enhancer factor 2A | MEF2A | 0.0372 |
| 293 | T-cell, immune regulator 1, ATPase, H+ transporting, lysosomal V0 | TCIRG1 | 0.0373 |
| 294 | phosphorylase kinase, alpha 1 (muscle) | PHKA1 | 0.0374 |
| 295 | epidermal growth factor receptor pathway substrate 8 | EPS8 | 0.0376 |
| 296 | Sel1 repeat containing 1 | SELRC1 | 0.0377 |
| 297 | homeobox A3 | HOXA3 | 0.0377 |
| 298 | protein phosphatase 1, regulatory subunit 37 | PPP1R37 | 0.0377 |
| 299 | jumonji domain containing 6 | JMJD6 | 0.0380 |
| 300 | stomatin | STOM | 0.0381 |
| 301 | blepharophimosis, epicanthus inversus and ptosis, candidate 1 | BPESC1 | 0.0381 |
| 302 | related RAS viral (r-ras) oncogene homolog 2 | RRAS2 | 0.0383 |
| 303 | chromosome 10 open reading frame 55 | C10orf55 | 0.0384 |
| 304 | ADAM metallopeptidase domain 21 | ADAM21 | 0.0384 |
| 305 | DNAJC25-GNG10 readthrough | DNAJC25-GNG10 | 0.0385 |
| 306 | lysyl oxidase | LOX | 0.0390 |
| 307 | solute carrier family 7 | SLC7A1 | 0.0390 |
| 308 | tubby like protein 1 | TULP1 | 0.0395 |
| 309 | epsin 2 2 | EPN2 | 0.0396 |
| 310 | solute carrier family 2 (facilitated glucose transporter) | SLC2A3 | 0.0396 |
| 311 | potassium channel tetramerisation domain containing 21 | KCTD21 | 0.0398 |
| 312 | EF-hand calcium binding domain 10 | EFCAB10 | 0.0398 |
| 313 | squamous cell carcinoma antigen recognized by T cells 3 | SART3 | 0.0399 |
| 314 | POM121 and ZP3 fusion | POMZP3 | 0.0400 |
| 315 | metastasis associated in colon cancer 1 | MACC1 | 0.0401 |
| 316 | doublesex and mab-3 related transcription factor 2 | DMRT2 | 0.0402 |
| 317 | nudix (nucleoside diphosphate linked moiety X)-type motif 9 | NUDT9 | 0.0402 |
| 318 | phosphatidylinositol 4-kinase type 2 beta | PI4K2B | 0.0403 |
| 319 | SHC SH2-domain binding protein 1-like | SHCBP1L | 0.0403 |
| 320 | olfactory receptor, family 2, subfamily A, member 4 | OR2A4 | 0.0403 |
| 321 | calcium channel, voltage-dependent, R type, alpha 1E subunit | CACNA1E | 0.0404 |
| 322 | glutaminyl-peptide cyclotransferase | QPCT | 0.0406 |
| 323 | premature ovarian failure, 1B | POF1B | 0.0406 |
| 324 | S-phase kinase-associated protein 2, E3 ubiquitin protein ligase | SKP2 | 0.0408 |
| 325 | colony stimulating factor 1 (macrophage) | CSF1 | 0.0414 |
| 326 | cytochrome P450, family 2, subfamily C, polypeptide 18 | CYP2C18 | 0.0415 |
| 327 | membrane-spanning 4-domains, subfamily A, member 3 | MS4A3 | 0.0415 |
| 328 | RANBP2-like and GRIP domain containing 5 | RGPD5 | 0.0416 |
| 329 | protein phosphatase 1, regulatory subunit 16A | PPP1R16A | 0.0417 |
| 330 | liver expressed antimicrobial peptide 2 | LEAP2 | 0.0418 |
| 331 | importin 5 | IPO5 | 0.0420 |
| 332 | CDV3 homolog (mouse) | CDV3 | 0.0427 |
| 333 | long intergenic non-protein coding RNA 242 | LINC00242 | 0.0428 |
| 334 | ectonucleotide pyrophosphatase/phosphodiesterase 3 | ENPP3 | 0.0428 |
| 335 | UDP-N-acetyl-alpha-D-galactosamine | GALNT2 | 0.0431 |
| 336 | eukaryotic translation initiation factor 4E family member 3 | EIF4E3 | 0.0433 |
| 337 | ADAM metallopeptidase domain 18 | ADAM18 | 0.0433 |
| 338 | killer cell immunoglobulin-like receptor, three domains | KIR3DL1 | 0.0434 |
| 339 | PR domain containing 15 | PRDM15 | 0.0434 |
| 340 | crystallin, gamma C | CRYGC | 0.0437 |
| 341 | digestive organ expansion factor homolog (zebrafish) | DIEXF | 0.0437 |
| 342 | leucine rich repeat containing 8 family, member D | LRRC8D | 0.0438 |
| 343 | RANBP2-like and GRIP domain containing 4 | RGPD4 | 0.0439 |
| 344 | solute carrier family 25 (pyrimidine nucleotide carrier) | SLC25A33 | 0.0440 |
| 345 | Tctex1 domain containing 2 | TCTEX1D2 | 0.0440 |
| 346 | acyl-CoA dehydrogenase family, member 10 | ACAD10 | 0.0441 |
| 347 | dynein, light chain, LC8-type 1 | DYNLL1 | 0.0442 |
| 348 | membrane-spanning 4-domains, subfamily A, member 2 | MS4A2 | 0.0443 |
| 349 | Nedd4 family interacting protein 2 | NDFIP2 | 0.0444 |
| 350 | olfactory receptor, family 10, subfamily AA, member 1 | OR10AA1P | 0.0446 |
| 351 | Src-like-adaptor | SLA | 0.0446 |
| 352 | pericentrin | PCNT | 0.0447 |
| 353 | solute carrier family 25, member 44 | SLC25A44 | 0.0447 |
| 354 | HEAT repeat containing 3 | HEATR3 | 0.0447 |
| 355 | tetratricopeptide repeat domain 12 | TTC12 | 0.0448 |
| 356 | NADP-dependent oxidoreductase domain containing 1 | NOXRED1 | 0.0449 |
| 357 | hematopoietic prostaglandin D synthase | HPGDS | 0.0449 |
| 358 | PRP3 pre-mRNA processing factor 3 homolog (S. cerevisiae) | PRPF3 | 0.0449 |
| 359 | phospholipase C, eta 1 | PLCH1 | 0.0450 |
| 360 | S100 calcium binding protein A12 | S100A12 | 0.0451 |
| 361 | Yip1 domain family, member 4 | YIPF4 | 0.0451 |
| 362 | phospholipid scramblase 4 | PLSCR4 | 0.0452 |
| 363 | olfactory receptor, family 6, subfamily K, member 3 | OR6K3 | 0.0454 |
| 364 | CD1d molecule | CD1D | 0.0456 |
| 365 | ARP2 actin-related protein 2 homolog (yeast) | ACTR2 | 0.0456 |
| 366 | ribonuclease, RNase K | RNASEK | 0.0458 |
| 367 | mitogen-activated protein kinase 3 | MAPK3 | 0.0459 |
| 368 | component of oligomeric golgi complex 8 | COG8 | 0.0462 |
| 369 | serine/threonine kinase 19 | STK19 | 0.0464 |
| 370 | charged multivesicular body protein 4A | CHMP4A | 0.0464 |
| 371 | tubulin, beta 4A class IVa | TUBB4A | 0.0467 |
| 372 | F-box protein 16 | FBXO16 | 0.0468 |
| 373 | Rho GTPase activating protein 23 | ARHGAP23 | 0.0470 |
| 374 | interferon, alpha-inducible protein 6 | IFI6 | 0.0470 |
| 375 | DiGeorge syndrome critical region gene 6-like | DGCR6L | 0.0471 |
| 376 | CD14 molecule | CD14 | 0.0472 |
| 377 | adaptor-related protein complex 5, beta 1 subunit | AP5B1 | 0.0472 |
| 378 | TAR (HIV-1) RNA binding protein 1 | TARBP1 | 0.0474 |
| 379 | forkhead box O1 | FOXO1 | 0.0475 |
| 380 | catenin (cadherin-associated protein), alpha 3 | CTNNA3 | 0.0475 |
| 381 | DAZ associated protein 1 | DAZAP1 | 0.0478 |
| 382 | phospholipase D family, member 3 | PLD3 | 0.0480 |
| 383 | ectonucleoside triphosphate diphosphohydrolase 6 (putative) | ENTPD6 | 0.0483 |
| 384 | interleukin 24 | IL24 | 0.0486 |
| 385 | pyruvate dehydrogenase kinase, isozyme 1 | PDK1 | 0.0487 |
| 386 | histidine decarboxylase | HDC | 0.0489 |
| 387 | fascin homolog 1, actin-bundling protein | FSCN1 | 0.0490 |
| 388 | alanyl-tRNA synthetase | AARS | 0.0490 |
| 389 | small nuclear ribonucleoprotein polypeptide E | SNRPE | 0.0490 |
| 390 | arachidonate 5-lipoxygenase-activating protein | ALOX5AP | 0.0490 |
| 391 | pallidin homolog (mouse) | PLDN | 0.0491 |
| 392 | chromosome 7 open reading frame 25 | C7orf25 | 0.0492 |
| 393 | Down syndrome critical region gene 6 | DSCR6 | 0.0492 |
| 394 | synaptopodin 2 | SYNPO2 | 0.0492 |
| 395 | tripartite motif containing 3 | TRIM3 | 0.0494 |
| 396 | migration and invasion enhancer 1 | MIEN1 | 0.0494 |
| 397 | protein kinase, AMP-activated, beta 1 non-catalytic subunit | PRKAB1 | 0.0494 |
| 398 | PRP6 pre-mRNA processing factor 6 homolog (S. cerevisiae) | PRPF6 | 0.0495 |
| 399 | CMT1A duplicated region transcript 1 | CDRT1 | 0.0496 |
| 400 | copine I | CPNE1 | 0.0497 |
| 401 | glutathione peroxidase 3 (plasma) | GPX3 | 0.0498 |
| 402 | HEAT repeat containing 6 | HEATR6 | 0.0498 |
| 403 | tetratricopeptide repeat domain 27 | TTC27 | 0.0498 |
| 404 | cystatin C | CST3 | 0.0499 |
| 405 | RNA binding motif protein, Y-linked, family 1, member B | RBMY1B | 0.0499 |
